# Genome-Wide Profiling of Histone Modifications (H3K9_me2_ and H4K12_ac_) and Gene Expression in Rust (*Uromyces appendiculatus*) Inoculated Common Bean (*Phaseolus vulgaris* L.)

**DOI:** 10.1371/journal.pone.0132176

**Published:** 2015-07-13

**Authors:** Vasudevan Ayyappan, Venu Kalavacharla, Jyothi Thimmapuram, Ketaki P. Bhide, Venkateswara R. Sripathi, Tomasz G. Smolinski, Muthusamy Manoharan, Yaqoob Thurston, Antonette Todd, Bruce Kingham

**Affiliations:** 1 Molecular Genetics and Epigenomics Laboratory, Delaware State University, Dover, Delaware, United States of America; 2 Center for Integrated Biological and Environmental Research (CIBER), Delaware State University, Dover, Delaware, United States of America; 3 Bioinformatics Core, Purdue University, West Lafayette, Indiana, United States of America; 4 Computational Intelligence and Bio(logical)informatics Laboratory (CIBiL), Delaware State University, Dover, Delaware, United States of America; 5 Department of Agriculture, University of Arkansas, Pine Bluff, Arkansas, United States of America; 6 Sequencing and Genotyping Center, Delaware Biotechnology Institute, Newark, Delaware, United States of America; Università degli Studi di Milano, ITALY

## Abstract

Histone modifications such as methylation and acetylation play a significant role in controlling gene expression in unstressed and stressed plants. Genome-wide analysis of such stress-responsive modifications and genes in non-model crops is limited. We report the genome-wide profiling of histone methylation (H3K9_me2_) and acetylation (H4K12_ac_) in common bean (*Phaseolus vulgaris* L.) under rust (*Uromyces appendiculatus*) stress using two high-throughput approaches, chromatin immunoprecipitation sequencing (ChIP-Seq) and RNA sequencing (RNA-Seq). ChIP-Seq analysis revealed 1,235 and 556 histone methylation and acetylation responsive genes from common bean leaves treated with the rust pathogen at 0, 12 and 84 hour-after-inoculation (hai), while RNA-Seq analysis identified 145 and 1,763 genes differentially expressed between mock-inoculated and inoculated plants. The combined ChIP-Seq and RNA-Seq analyses identified some key defense responsive genes (calmodulin, cytochrome p450, chitinase, DNA Pol II, and LRR) and transcription factors (WRKY, bZIP, MYB, HSFB3, GRAS, NAC, and NMRA) in bean-rust interaction. Differential methylation and acetylation affected a large proportion of stress-responsive genes including resistant (R) proteins, detoxifying enzymes, and genes involved in ion flux and cell death. The genes identified were functionally classified using Gene Ontology (GO) and EuKaryotic Orthologous Groups (KOGs). The Kyoto Encyclopedia of Genes and Genomes (KEGG) pathway analysis identified a putative pathway with ten key genes involved in plant-pathogen interactions. This first report of an integrated analysis of histone modifications and gene expression involved in the bean-rust interaction as reported here provides a comprehensive resource for other epigenomic regulation studies in non-model species under stress.

## Introduction

Plants are sessile organisms that cannot physically relocate to escape from unfavorable environmental conditions and have developed complex defense mechanisms to respond to biotic and abiotic stresses. The molecular mechanisms of stress-induced signaling pathways and genes differ between various stresses such as pathogen attack, cold, heat, drought, and salinity [1, 2]. However, there is supporting evidence for cross talk between signaling pathways that respond to biotic and abiotic stresses [3]. Pathogen stress is one of the most complex and devastating stresses that is witnessed in plants [4]. Pattern-triggered immunity (PTI) is activated in plants as an early defense response [5] and the role of defense-related genes including cell wall modifying genes has been reported [6].

In agriculturally important crops such as common bean, significant yield losses due to biotic (62%) and abiotic (37–67%) stresses have been reported [7]. Bean-rust, a disease caused by the fungal pathogen *Uromyces appendiculatus* is a major concern for common bean production worldwide [8]. Tropical and subtropical areas in the world have been mostly affected by epidemics of this disease. The diversity in virulence of *U*. *appendiculatus* in many geographic regions has been reported [9–11]. The high genetic variability of the rust fungus is an important problem that continues to complicate the development of durable resistant varieties in common bean. Integrated molecular genetic and genomic analyses of defense responsive pathways and genes will aid in unraveling the underlying disease-resistance mechanisms, which in turn will aid in developing broader and more robust resistance in common bean cultivars while providing a more comprehensive understanding of plant disease resistance in general.

Epigenetic and epigenomic regulation including histone and chromatin modifications can modulate stress responses by activating or repressing transcription by coordinating “open” or “closed” chromatin conformations, respectively [12]. In some cases, chromatin changes are steady and autonomous as a result of heritable epialleles that induce phenotypic alteration [13]. Epigenetic modifications can be induced by sustained exposure to pathogens that result in a stable epigenetic characteristic of resistance or tolerance [14]. Changes in histone modification marks have been shown to influence gene regulatory mechanisms in *Arabidopsis thaliana* [15]. Diversity in gene expression at both the tissue-specific and population levels has been reflected by the alteration of DNA methylation [4]. Determining the role of transcriptional networks is not only helpful in understanding the molecular mechanisms of plant responses to biotic and abiotic stress tolerance, but it is also useful for improving stress tolerance by genetic engineering. Previous studies showed that histone modifications are involved in abiotic [16, 17] and biotic responses [18, 19]. In transgenic *Arabidopsis*, over-expression of the histone deacetylase, AtHD2C resulted in abscisic acid (ABA) insensitivity and showed tolerance to salt and drought stresses [20]. The histone acetyltransferase1-dependent epigenetic mark involved in pattern triggered immunity (PTI) against *Pseudomonas syringae* has been reported in *Arabidopsis* [18]. In biotic and abiotic stresses, plants orchestrate gene expression in coordination with several proteins or transcription factors (TFs) [21]. Transcription factors up- or down-regulate the expression of stress responsive genes independently or by recruiting other associated proteins [22].

Significant success has been achieved in developing stress-tolerant varieties by utilizing traditional plant breeding methodologies [23]. More recently, next generation sequencing (NGS) technologies have been employed to better understand the defense responsive genes, proteins and regulatory elements involved in various metabolic pathways in plants such as *Arabidopsis* [24], rice [25], peas [26] and beans [27]. During stress, plants defend themselves by modulating genome-wide gene expression associated with various physiological functions [28]. Such genome-wide expression and regulation studies are feasible with the advent of high-throughput technologies including ChIP-Seq and RNA-Seq. Due to the sensitivity and specificity of ChIP-Seq technology in identifying protein-DNA interactions, it has been used in generating high-resolution epigenomic maps in plants including *Arabidopsis* [29], rice [30], and maize [31]. ChIP-Seq has been extensively used on large mammalian genomes to map *in vivo* transcription factor (TF)-binding sites [32], and histone marks [33]. RNA-Seq has also been extensively utilized in analyzing genome-wide gene expression including coding and non-coding RNAs as demonstrated in many studies [34–36]. RNA-Seq provides a comprehensive analysis of over-expressed and under-expressed genes with minimal bias when compared to microarray analysis [37]. Furthermore, RNA-Seq analysis was successfully utilized in analyzing gene expression from different organs under different treatment conditions in plants and to identify gene homologs [27, 38]. Many reports are also available on transcriptome-wide analysis in plants in response to biotic [38] and abiotic stresses [39]. However, until now, no comprehensive report is available in understanding gene expression and regulation of the common bean-rust interactions in combination with either histone or DNA modifications. This study, therefore is aimed at understanding epigenomic and transcriptomic changes in common bean challenged with fungal rust, *U*. *appendiculatus* using ChIP-Seq and RNA-Seq.

## Results and Discussion

To understand differential histone DNA binding and transcriptional regulation of genes involved in the bean-bean rust race 53 interaction, we constructed ChIP-Seq and RNA-Seq libraries from inoculated (I) and mock-inoculated (MI) leaves of the bean-rust resistant cultivar, “Sierra”. The experiment included three biological replicates collected at three different time points (0, 12 and 84 hours-after-inoculation, hai) and two treatment conditions (rust-inoculated, I and mock-inoculated, MI). The libraries utilized in this study were labeled as 0I, 0MI, 12I, 12MI, 84I and 84MI (Table 1). Additionally, the same experimental material was used in generating both the ChIP-Seq and RNA-Seq libraries and sequenced on Illumina/HiSeq-2500 platform to understand meaningful relationships between genome-wide histone-DNA binding and regulation of gene expression.

**Table 1:** Summary statistics of RNA-Seq and ChIP-Seq reads (Illumina/HiSeq2500) collected from three biological replicates of rust-inoculated and mock-inoculated common bean.

Library		# of reads collected			# of reads after adapter Trimming			# of reads mapped to the reference genome			% of mapped reads		
Type	Time points	R1	R2	R3	R1	R2	R3	R1	R2	R3	R1	R2	R3
**RNA-Seq**	**0I**	17017033	28661262	28672060	17000220	28634250	28647560	14190992	26701910	27019003	83.50	93.30	94.30
	**0MI**	33534366	25995784	24825109	33504428	25968064	24804585	32044658	24655466	23024657	95.60	94.90	92.80
	**12I**	21718196	29372600	20465199	21701589	29343958	20450429	20502613	27918863	18422943	94.50	95.10	90.10
	**12MI**	23108163	34974631	25843144	23091650	34938134	25824894	21941936	32647639	23770607	95.00	93.40	92.00
	**84I**	27755126	31309689	20150530	27733102	31277816	20135631	26319460	29829345	18970455	94.90	95.40	94.20
	**84MI**	27627616	30307173	18229334	27600121	30278582	18215555	26087868	28877706	17142924	94.50	95.40	94.10
**ChIP-Seq/ H3K9**_**me2**_	**0I**	33935039	9087019	12569880	31444528	8545776	12161003	25867409	6827317	10337785	82.26	79.89	85.01
	**0MI**	25841364	8287431	12207298	24516929	7988480	11449028	18774232	6406975	9867138	76.58	80.20	86.18
	**12I**	20816533	12681234	10466318	18440162	11977627	9966856	15454953	9134265	8212035	83.81	76.26	82.39
	**12MI**	22130415	9624108	10409303	17873334	9089221	10036112	11616873	7136289	7746010	66.75	78.51	77.18
	**84I**	21240492	7389011	9912237	19129702	7089343	9440740	16121256	5661474	7348053	84.27	79.86	77.83
	**84MI**	25038216	9461436	9825009	20225242	9043213	9449445	15456484	6961811	7826139	79.98	76.98	82.82
**ChIP-Seq/ H4K12**_**ac**_	**0I**	7979252	8912037	7740349	7447801	6136091	7441667	5714157	4755505	6293474	76.72	77.50	84.57
	**0MI**	10767322	8902952	9684972	10229264	8546571	9250423	7911502	6913545	7938609	77.34	80.89	85.82
	**12I**	14079044	8456181	8414951	12930437	8151328	7903373	9464610	6088243	6303431	73.20	74.69	79.76
	**12MI**	15691673	12216382	10827028	14898905	10807095	10156112	10420441	7743696	8760141	69.94	71.65	86.25
	**84I**	19187656	9669249	8694177	18326579	9335039	8381921	13777024	7317725	6737029	75.18	78.39	80.38
	**84MI**	13496314	9272589	9344326	12877810	8869909	8860367	9640994	7058824	6622265	74.39	79.58	74.74

### Data collection and pre-processing

In total, we generated 54 libraries to undertake RNA-Seq and ChIP-Seq analyses in common bean. For RNA-Seq analysis, 18 libraries were prepared from the leaf samples collected from three biological replicates, two treatment conditions (inoculated and mock-inoculated) and three collection time points after inoculation (3 biological replicates x 2 treatment conditions x 3 collection time points after treatment = 18). Similarly, in ChIP-Seq analysis, for each histone mark (H3K9_me2_ and H4K12_ac_), 18 libraries each were prepared exactly from the same sample source that was utilized in RNA-Seq analysis to maintain uniform experimental conditions, thus resulting in 36 ChIP-Seq libraries with two histone marks, H3K9_me2_ and H4K12_ac_. Moreover, in this study mock-inoculated (MI) samples served as background (similar to no antibody in other studies) against inoculated samples, which was used for the comparison during ChIP-Seq analysis to identify differentially marked regions. Deep sequencing of 54 libraries in common bean resulted in ~933 million-50 bp Illumina reads (Table 1). Of these, ~469 million reads were from RNA-Seq and the rest (~464 million reads) were from ChIP-Seq. Within the ChIP-Seq reads, ~271 and ~193 million reads were derived from H3K9_me2_ (methylation) and H4K12_ac_ (acetylation) histone marks, respectively. The relatively lower number of reads in ChIP-Seq, when compared to RNA-Seq is due to the limited specificity of acetylated and methylated histone binding across the genome. The raw reads obtained from ChIP-Seq and RNA-Seq were trimmed, filtered, and high quality reads thus collected were aligned to the *P*. *vulgaris* G19833 genome (V1.0, accessed 07 August 2012) from Phytozome [40]. Over 75% of the ChIP-Seq reads and >95% of the RNA-Seq reads were mapped to the reference genome and only uniquely mapped reads with ≤ 2 mis-matches were further used in analysis. The details of ChIP-Seq and RNA-Seq analyses with mapping statistics are presented in Table 1.

### Histone marks identified in common bean-rust interaction

**Fig 1:**
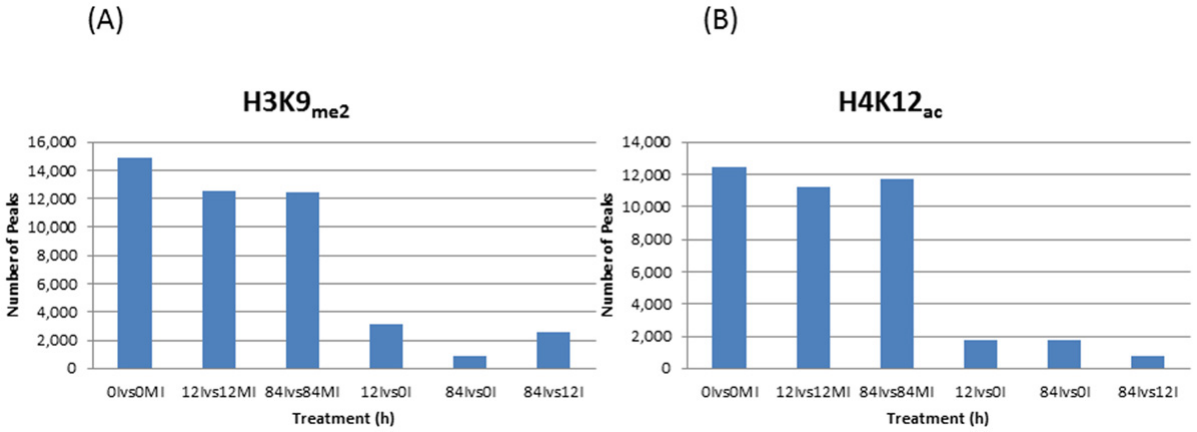
Identification of (A) H3K9_me2_ and (B) H4K12_ac_ regions in mock-inoculated and inoculated common bean. The number of peaks at 0, 12 and 84 hai were compared against their respective backgrounds, 0MI, 12MI and 84MI. The peaks were also compared between 12Ivs0I, 84Ivs0I and 84Ivs12I against two histone modifications.

In ChIP-Seq analysis, peak calling is very critical and this study utilized Spatial Clustering for Identification of ChIP-Enriched Regions (SICER) [41] to identify 14,857, 12,521, and 12,448 peaks for H3K9_me2_ and 12,426, 11,205 and 11,724 peaks for H4K12_ac_ when 0I, 12I, and 84I were compared against their respective backgrounds, 0MI, 12MI and 84MI (Fig 1). Also we compared the number of regions that were differentially marked between these time points (0, 12 and 84 hai) independently against two histone modifications. The majority of the differentially marked regions were represented in at least two out of three replicates being investigated. For the methylation (H3K9_me2_) mark, we identified 3,100 (12Ivs0I), 838 (84Ivs0I), and 2,603 (84Ivs12I) differentially marked regions between time points, respectively (Fig 1A). For the acetylation (H4K12_ac_) mark, the differentially marked regions (peaks) identified between the time periods include: 1,757 (12Ivs0I), 1,714 (84Ivs0I) and 808 (84Ivs12I) (Fig 1B). The number of peaks identified was lower between the time points when compared to the background, suggesting the specificity of the peaks to each treatment point.

Our next goal was to identify the nature of the regions associated with the differentially marked peaks in the genome for both the H3K9_me2_ and H4K12_ac_ marks. We utilized Hypergeometric Optimization of Motif EnRichment (HOMER) [42] for annotating the peaks into: Exon, Intron, Promoter, Transcription Termination Sites (TTS), and Intergenic regions on the basis of annotation of known genes. We considered a peak as ‘genic’ only if it lies between the start and end position of a gene, which include 5’ and 3’ UTRs but not promoter regions while the region between two genes is treated as inter-genic region. However, the center of the peak position primarily determines the nature of the peak. Mainly, our annotation included ‘promoter-TSS’ as the region between -1Kb to +100bp and ‘TTS’ as the region between -100bp to + 1Kb, while the introns, exons and intergenic regions were directly utilized from annotation information. In our study, more peaks (>70%) were identified in the intergenic regions than in genic regions (<18%) across all the time points (Table 2). Our study is in accordance with previous reports in mammalian studies [43], which showed a similar distribution of H4K12_ac_ and H3K9_me2_ marks across genic and intergenic regions. Additionally, this work concurs with a study in rice, where ~83% of the reads aligned to intergenic regions with only few reads aligning to genic locations [44].

**Table 2:** Summary of peaks annotated, features extracted, and number of peaks identified per feature in common bean.

		H3_12Ivs0I		H3_84Ivs0I		H3_84Ivs12I		H4_12Ivs0I		H4_84Ivs0I		H4_84Ivs12I	
Sub-regions on the genome	Total size (bp)	Number of peaks	% of total peaks	Number of peaks	% of total peaks	Number of peaks	% of total peaks	Number of peaks	% of total peaks	Number of peaks	% of total peaks	Number of peaks	% of total peaks
**TTS**	26956527	20	0.65	17	2.03	29	1.11	95	5.41	71	4.14	61	7.55
**Exon**	37848497	27	0.87	25	2.98	23	0.88	30	1.71	34	1.98	34	4.21
**Intron**	56192331	46	1.48	31	3.70	31	1.19	69	3.93	51	2.98	49	6.06
**Intergenic**	363765093	2978	96.06	741	88.42	2490	95.66	1489	84.75	1506	87.86	598	74.01
**Promoter-TSS**	29853573	29	0.94	24	2.86	30	1.15	74	4.21	52	3.03	66	8.17

**Fig 2:**
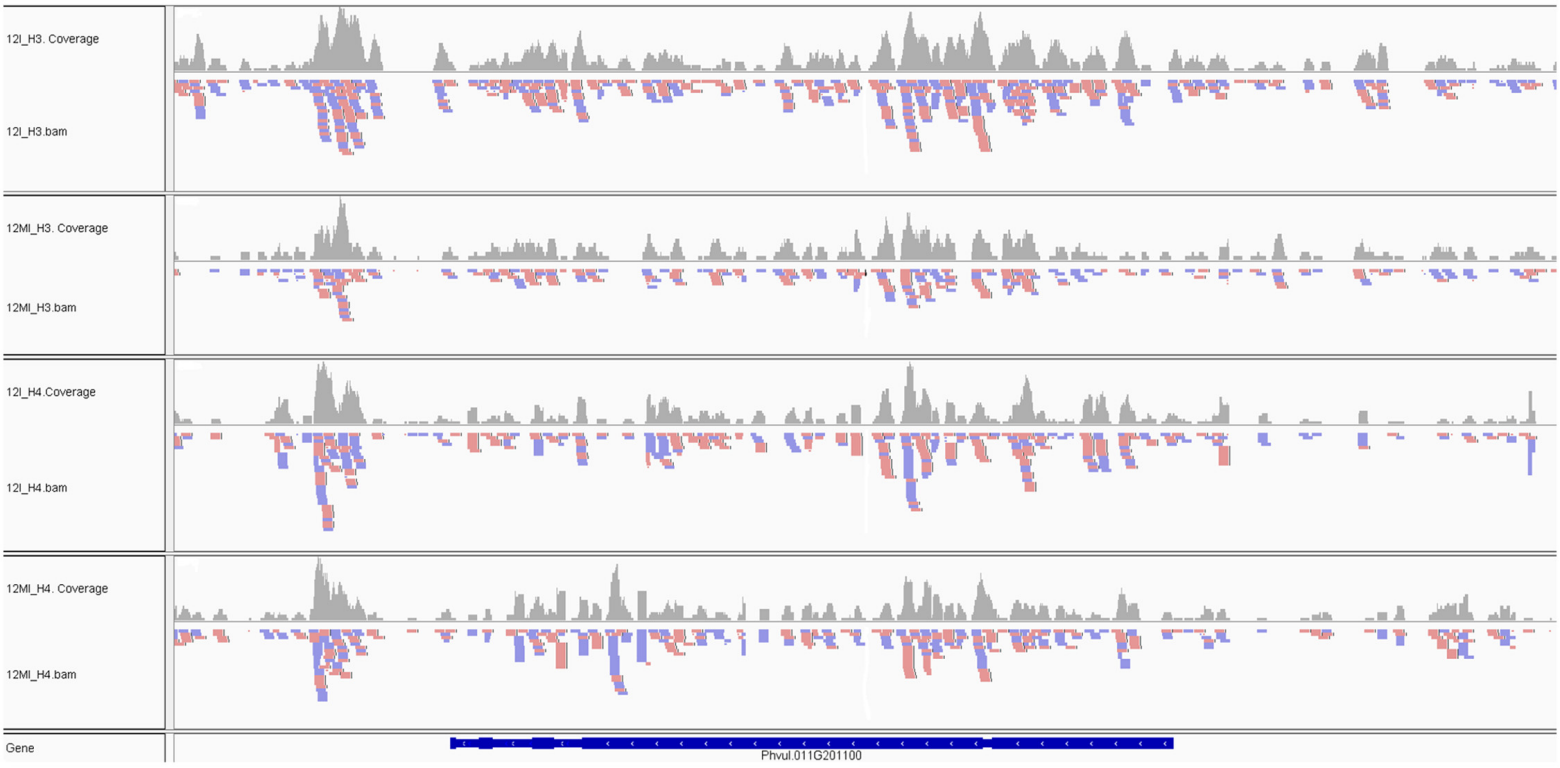
Peak signals visualized on common bean chromosomes by using Genome Viewer. Comparative visualization of a representative region on chromosome 11 in rust-inoculated common bean at 12 hai with mock-inoculated sample as background for H3K9_me2_ and H4K12_ac_ modifications using Integrative Genomics Viewer (IGV).

We next assigned the histone-DNA-binding locations to eleven chromosomes. The highest number of H3K9_me2_ marks were seen on chromosome (chr) 11 followed by chr07, 10, 08 and 05 while the H4K12_ac_ marks were seen on chr11 followed by chr03, 01 and 02 (S1 Table). The majority of differential histone methylation and histone acetylation marks were found between the time points 12 and 0 hai, suggesting that more differentially marked regions were identified in early inoculation. On the other hand, 84Ivs12I had maximum number of genes marked in chr11 followed by chr7 and chr10 (S1 Table). The least number of overlapping genes were identified on chr06. Further, the distribution of histone marks on 11 chromosomes were manually visualized using the Integrative Genomics Viewer (IGV) genome browser [45] (Fig 2).

### Genes associated with epigenetic regulation during common bean-rust interaction

The RNA-Seq and ChIP-Seq reads were aligned separately to the reference genome (G19833) and then compared to understand global gene expression and epigenetic regulation in rust-infected common bean that identified 279, 45, 145, 26 and 225 genes associated with DNA methylation, histone methylation, histone acetylation, chromatin remodeling, and Polycomb Group (PcG) proteins, respectively (S1 Fig). The majority (70%) of the genes identified belonged to DNA methylation and PcG proteins, which play a critical role in transcriptional regulation in addition to DNA stability. Further, we analyzed genes coding for histone modifications and identified more histone methylation related genes (39%) than histone acetylation-associated genes (20%). The preferential binding of histone methylation marks to common bean chromosomes was observed when compared to histone acetylation marks, suggesting that the gene activity is selectively regulated in common bean-rust interaction.

Histone lysine methylation regulates chromatin function and epigenetic control of gene expression. The methylation mark identified Su(var)3-9, Enhancer-of-zeste, Trithorax (SET) domain binding proteins, polyamine oxidases (-1, -2, and -4), phytoene desaturase 3, zeta-carotene desaturase, homocysteine S-methyltransferase family protein, and lysine specific demethylase (LSD) family proteins. However, the SET domain binding (30) and LSD family of proteins (6) that are commonly associated with histone lysine methylation were preferentially marked by H3K9_me2_. Histone acetylation plays an important role in modulating chromatin structure and function by adding or removing acetyl groups to the conserved N-terminal lysines of histones [46]. Acetylation is generally associated with transcriptional activation and several HATs have been identified as transcriptional co-activators. In animals, high levels of trimethylation (H3K4_me3_) have been associated with the recruitment of chromatin remodeling factors and other effector proteins in pathogen primed samples [47, 48]. The differentially expressed histone binding and chromatin remodeling genes identified include Alfin-like 6, like heterochromatin protein 1 (LHP1), chromatin assembly factor, and chromatin remodeling factors (crf), crf-18, crf-8, and crf-24, which aid in repositioning of nucleosomes associated with stress.

### Resistance-related genes marked by methylation and acetylation

Plants respond to microbial pathogens by systematically eliciting the hypersensitive response (HR) immediately after detection of the pathogenesis factor by plant disease resistance related proteins. In support of this, we identified four HR-related genes (HR-like lesion inducing proteins) that may have possible roles in common bean-rust HR-signaling. The roles of several plant resistance (R) proteins in fungal pathogen recognition and eliciting immune responses have been reported [49]. The methylation modification with H3K9_me2_ was predominantly bound to disease resistance family of proteins including the leucine rich repeat (LRR) family, NB-ARC domain containing and Toll Interleukin Receptor-Nucleotide Binding Site-Leucine Rich Repeat (TIR-NBS-LRR) class of proteins that were located on chr01, chr07, chr08, chr10 and chr11 (Table 3). Meanwhile H4K12_ac_ was seen to target only NB-ARC domain containing disease resistance proteins located on chr11 (Table 4). The role of NB-ARC domain-containing proteins have been implicated in stress [50]. Structurally, the ARC domain contains three elements, APAF-1 (apoptotic protease-activating factor-1), R proteins, and CED-4 (*Caenorhabditis elegans* death-4 protein). The NB-ARC, a functional ATPase domain with its nucleotide-binding (NB) site regulates the activity of R proteins [50].

**Table 3:** Difference in marking of disease-resistance genes by H3K9_me2_ modification at various time points after inoculation.

Gene ID	Description	ChIP-Seq-H3 (LogFC)		
		12Ivs0I	84Ivs0I	84Ivs12I
**Phvul.005G173700**	Pleiotropic Drug Resistance Protein 12	0.81–1.44	-	0.52–1.03
**Phvul.008G071300**	NB-ARC Domain Containing Disease Resistance Protein	0.66–0.87	0.78–0.80	0.96
**Phvul.001G226300**	Chaperone DnaJ-Domain Superfamily Protein	1.14–1.54	1.15–1.27	0.82–1.00
**Phvul.001G103300**	MATE Effluse Family Protein	0.68–1.03	0.67–0.72	1.00
**Phvul.001G042900**	Disease Resistance Family Protein/LRR Family Protein	1.12–1.52	-	0.58–0.98
**Phvul.007G246600**	Disease Resistance Family Protein/LRR Family Protein	0.94–1.05	0.71	0.82
**Phvul.010G027200**	Disease Resistance Protein (TIR-NBS-LRR Class) Family	0.66–1.21	0.66	0.75
**Phvul.010G063000**	NB-ARC Domain-Containing Disease Resistance Protein	0.72–0.90	0.82–0.95	0.66
**Phvul.011G201000**	LRR and NB-ARC Domain-Containing Disease Resistance Protein	0.66–1.33	0.62	0.65–0.84
**Phvul.011G201100**	NB-ARC Domain-Containing Disease Resistance Protein	0.66–1.33	0.62	0.65–0.84

**Table 4:** Difference in marking of disease-resistance genes by H4K12_ac_ modification at various time points after inoculation.

Gene ID	Description	ChIP-Seq-H4 (LogFC)		
		12Ivs0I	84Ivs0I	84Ivs12I
**Phvul.005G173700**	Pleiotropic Drug Resistant Protein 12	1.10	1.21	1.12–1.52
**Phvul.010G040400**	Multidrug Resistance Associated Protein 9	0.84–1.26	-	0.78–0.90
**Phvul.001G115100**	Leucine-rich Repeat Protein	0.85–1.21	0.62–1.06	0.78–0.88
**Phvul.011G195900**	NB-ARC Domain Containing Disease Resistance Protein	0.64–1.49	-	0.78
**Phvul.011G201100**	NB-ARC Domain Containing Disease Resistance Protein	1.50–2.24	-	0.39–0.78
**Phvul.001G042800**	Disease Resistance Family Protein/LRR Family Protein	0.41	0.51	-
**Phvul.011G151300**	NB-ARC Domain Containing Disease Resistance Protein	1.46	-	0.78
**Phvul.002G323400**	Disease Resistance Protein (TIR-NBS-LRR class)	2.24	-	0.92
**Phvul.010G091500**	Disease Resistance Protein (CC-NBS-LRR class)	0.91	-	0.66

The R proteins that were marked by both methylation and acetylation modifications include pleiotropic drug resistant protein 12, LRR family, NB-ARC domain containing, and TIR-NBS-LRR proteins (Table 3A and 3B). The other R proteins that were uniquely marked by acetylation include multi-drug resistant associated protein 9 and Coiled-Coil (CC) domain containing NBS-LRR protein while DnaJ-domain superfamily and Multi-antimicrobial extrusion protein (MATE) effluse family proteins were uniquely marked by methylation. The DnaJ-domain superfamily proteins that participate in cellular stress and protein biosynthesis were commonly represented across all three time points. The MATE-family transporter and ELKS/RAB6-interacting/CAST family member 1 (Erc1) that confers resistance to ethionine, an analog of methionine was reported in *Arabidopsis*-fungal interaction [51]. Similarly, we identified a MATE effluse family protein that may have a role in regulating carbon metabolism and mediating defense responses in bean-rust interaction. Differences in expression of R genes was compared across time points (0, 12 and 84I hai), and a maximum difference in fold change (>1) was observed at 12 hai, supporting the evidence that HR-signaling cascades were more active in early (12 hai) inoculation than in later inoculation (84 hai).

### Differences in gene expression during common bean-rust interaction

**Fig 3:**
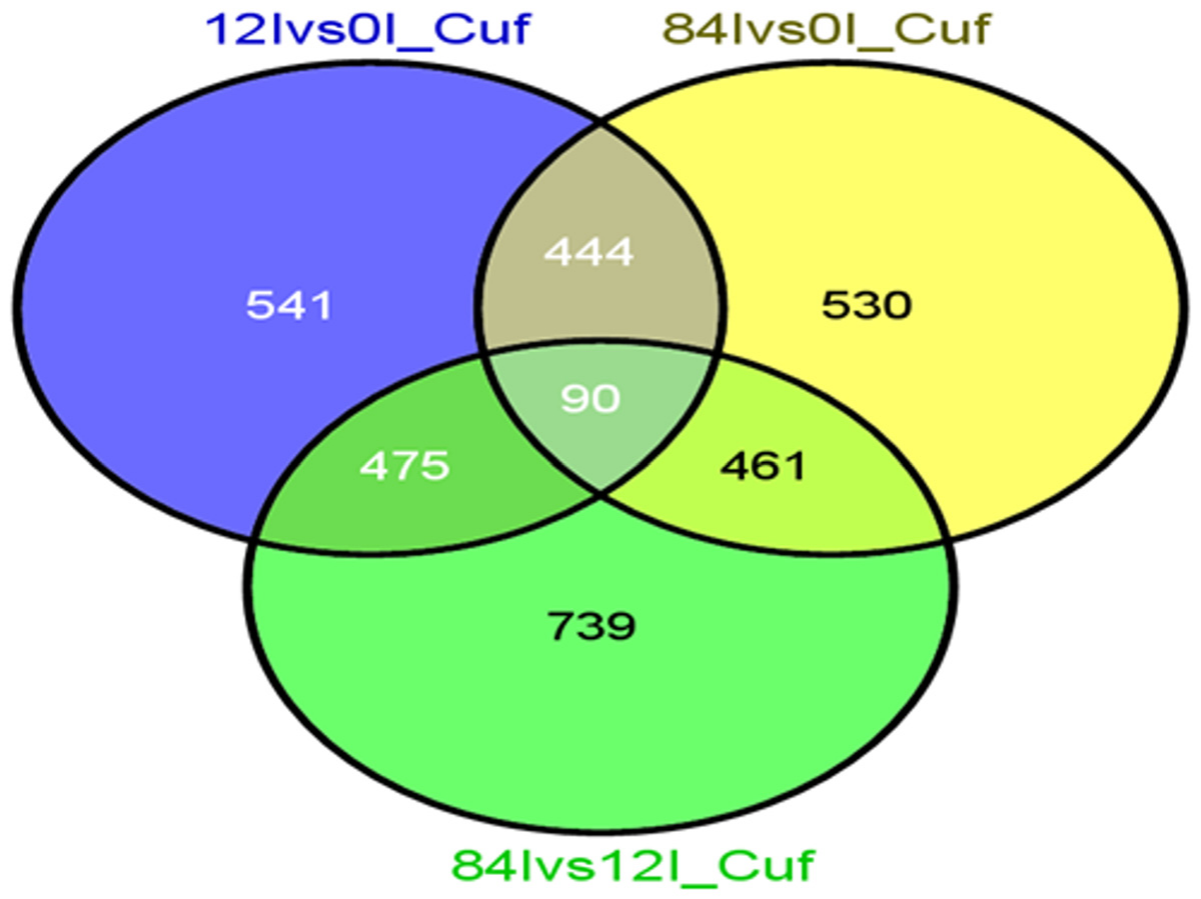
Total number of significantly enriched genes identified between different time points (0, 12 and 84 hai) in rust-inoculated common bean from RNA-Seq analysis. The number of uniquely and commonly enriched significant genes and their respective time points were depicted as a Venn diagram. For instance, the uniquely enriched significant genes and their respective time points include: 541 (12Ivs0I), 530 (84Ivs0I) and 739 (84Ivs12I).

RNA-Seq analysis identified more than 80% of the transcript derived reads as predicted genes. The differential expression analysis utilizing Cufflinks identified 1,369, 1,308 and 1,493 differentially expressed genes, and 541, 530 and 739 uniquely expressed genes between 12Ivs0I, 84Ivs0I, and 84Ivs12I, respectively (S2 Table). Among the differentially expressed genes, 90 were commonly shared between the three time points (Fig 3). The maximum differential expression was observed in four stress responsive genes including early-responsive to dehydration (ERD) stress family protein, chloroplast drought-induced stress protein, oxidative stress 3, and stress induced alpha-beta barrel domain protein between 0, 12 and 84 hai (Table 5). Oxidative stress 3 gene was up-regulated at 12 hai and down-regulated at 84 hai, supporting the evidence of ion flux during pathogenesis. The changes observed in ERD family and stress-induced alpha-beta barrel domain proteins across the three time points were provided (Table 5).

**Table 5:** Summary of differentially expressed stress-related genes at 12Ivs0I, 84Ivs0I and 84Ivs12I from RNA-Seq analysis.

Gene ID	Description	Log_2_FC		
		12Iv0I	84Ivs0I	84Ivs12I
**Phvul.004G174600**	Oxidative stress 3	1.44	-3.40	-4.84
**Phvul.006G085800**	Oxidative stress 3	0.59	-4.46	-5.05
**Phvul.002G307900**	Oxidative stress 3	1.44	-2.28	-3.73
**Phvul.011G091300**	Stress Responsive A/B Barrel Domain	3.34	2.31	-1.03
**Phvul.005G030600**	Early Responsive to Dehydration Stress Protein (ERD4)	-0.74	2.46	3.20
**Phuvl.001G148800**	ERD Family Protein Early-Responsive to Dehydration Stress	-0.84	-3.50	-2.72
**Phvul.002G321800**	Chloroplast Drought-Induced Stress Protein of 32KD	-1.13	-4.50	-3.36
**Phvul.001G263200**	ERD (Early Responsive to Dehydration Stress) Family Protein	-2.84	0.64	3.49
**Phvul.011G069900**	Oxidative Stress 3	2.78	0.24	-2.53

Besides stress-induced proteins, the peroxidase superfamily proteins were significantly enriched in our analysis. Based on the evolutionary and functional relationships within the plant peroxidase superfamily proteins, three structurally diverse classes have been reported. In this study, we identified secretory fungal peroxidases or class II superfamily of proteins that are implicated in the disruption of primary cell wall components including lignocelluloses and lignins. These proteins have suggested roles in fungal aspersorium formation and haustorium establishment. Moreover, the cell wall modifying enzymes were significantly expressed at 12 hai. Also, about 50 differentially expressed defense-related proteins identified were provided (Table 6). Among these, Late elongated hypocotyl (LHY), Ethylene Response Factor (ERF), W box containing TF (WRKY), and protodermal factor (PDF) proteins were abundantly represented in the dataset and differentially expressed between the time points (0, 12 and 84 hai).

**Table 6:** Summary of defense-related genes from RNA-Seq analysis.

Gene ID	Description	Log_2_FC		
		12Ivs0I	84Ivs0I	84Ivs12I
**Phvul.008G036200**	Defense No Death 1 (DND1)	1.04	-1.03	2.45
**Phvul.007G096300**	Phenylpropanoid Biosynthesis SA Biosynthesis (PAL1)	-0.67	1.89	2.56
**Phvul.006G169400**	Late Elongated Hypocotyl (LHY)	-3.36	-1.64	1.72
**Phvul.007G156000**	Late Elongated Hypocotyl (LHY)	-2.28	-0.48	1.79
**Phvul.010G120400**	Late Elongated Hypocotyl (LHY)	2.50	3.06	0.56
**Phvul.009G018300**	Late Elongated Hypocotyl (LHY)	3.18	4.25	1.07
**Phvul.007G182500**	Late Elongated Hypocotyl (LHY)	-4.20	-3.84	0.35
**Phvul.007G217800**	Ethylene Responsive Element Binding Protein (ATEBP)	4.30	0.26	-4.03
**Phvul.003G021000**	Jasmonate-zim-Domain Protein 3 (JAZ3)	2.02	2.64	0.62
**Phvul.011G071400**	Mitogen-Activated Protein Kinase 3 (MPK3)	1.06	-1.48	-2.54
**Phvul.006G204500**	RPMI-Interacting Protein 4 Family protein (RIN4)	-3.42	-1.65	1.77
**Phvul.008G279500**	RPMI-Interacting Protein 4 Family Protein (RIN4)	-3.64	1.53	5.18
**Phvul.004G071700**	Mildew Resistance Locus O Protein 12 (MLO12)	2.00	2.33	0.33
**Phvul.005G136700**	Mildew Resistance Locus O Protein 6 (MLO6)	4.77	0.72	-4.04
**Phvul.006G146400**	Chitin Elicitor Receptor Kinase 1 (CERK1)	-6.49	-3.27	3.22
**Phvul.008G211200**	Chitin Elicitor Receptor Kinase 1 (CERK1)	-3.22	-0.37	2.84
**Phvul.003G131500**	OPDA-Reductase 3 (OPR3)	-3.23	-1.67	1.58
**Phvul.011G049600**	Allene Oxidase Synthase (AOS)	-2.13	-1.39	0.73
**Phvul.007G055700**	Allene Oxidase Synthase (AOS)	-5.60	-2.22	7.38
**Phvul.007G193300**	ETH Response Factor 1 (ERF1)	2.26	-3.32	-5.58
**Phvul.007G217800**	ETH Response Factor 1 (ERF1)	3.36	-0.28	-3.64
**Phvul.007G193800**	ETH Response Factor 1 (ERF1)	-4.77	-4.38	0.39
**Phvul.006G183200**	ETH Response Factor 1 (ERF1)	-2.01	-1.59	0.41
**Phvul.001G160500**	ETH Response Factor 1 (ERF1)	3.56	1.45	-2.1
**Phvul.008G092800**	ERF48	4.14	0.16	-3.98
**Phvul.009G138900**	WRKY 51	2.77	-0.91	-3.69
**Phvul.008G081800**	WRKY 70	3.15	0.43	0.15
**Phvul.001G088200**	WRKY 75	3.76	1.47	-2.28
**Phvul.002G240900**	WRKY 65	-6.42	-1.35	5.07
**Phvul.009G080000**	WRKY 50	-3.89	2.37	6.27
**Phvul.008G270500**	WRKY 6	-3.78	0.94	4.72
**Phvul.006G047300**	WRKY 45	-3.45	-0.94	2.51
**Phvul.009G195200**	WRKY 32	-2.03	-0.84	1.19
**Phvul.002G196200**	Flagellin-Sensitive-2 (FLS2)	7.59	2.29	-5.29
**Phvul.002G081500**	Soluble N-Ethylmaleimide-Sensitive Factor Adaptor Protein 30 (SNAP 30)	-0.70	2.26	2.96
**Phvul.002G286500**	Osmotin 34	-2.46	1.65	4.11
**Phvul.002G286600**	D Peroxin 3	-4.95	2.94	7.90
**Phvul.002G155400**	Phosuducin-like Protein 3 Homolog	-2.29	1.33	3.63
**Phvul.003G116900**	ETH INSENSITIVE 2 (EIN2)	-2.31	1.15	3.47
**Phvul.002G202400**	MAP KINASE KINASE KINASE 1 (MEKK1)	1.51	-1.07	-2.58
**Phvul.009G158000**	PDF 2.5 (Protodermal Factor 2.5)	-3.21	-2.81	0.40
**Phvul.002G048200**	PDF 2	3.31	0.56	-2.75
**Phvul.005G071400**	PDF 2.3	-0.76	2.35	3.11
**Phvul.006G033900**	PDF 1	-0.84	2.95	-1.20
**Phvul.005G071300**	PDF 2.3	-0.21	2.51	2.73
**Phvul.007G053200**	PDF2	-1.51	-3.63	-2.34
**Phvul.006G137300**	RFO1, WAKL22 (Wall Associated Kinase Family Protein)	2.46	-0.29	-2.75
**Phvul.002G140900**	AIG2-like (Avirulence induced gene) Family Protein	-2.34	-0.05	2.29

**Fig 4:**
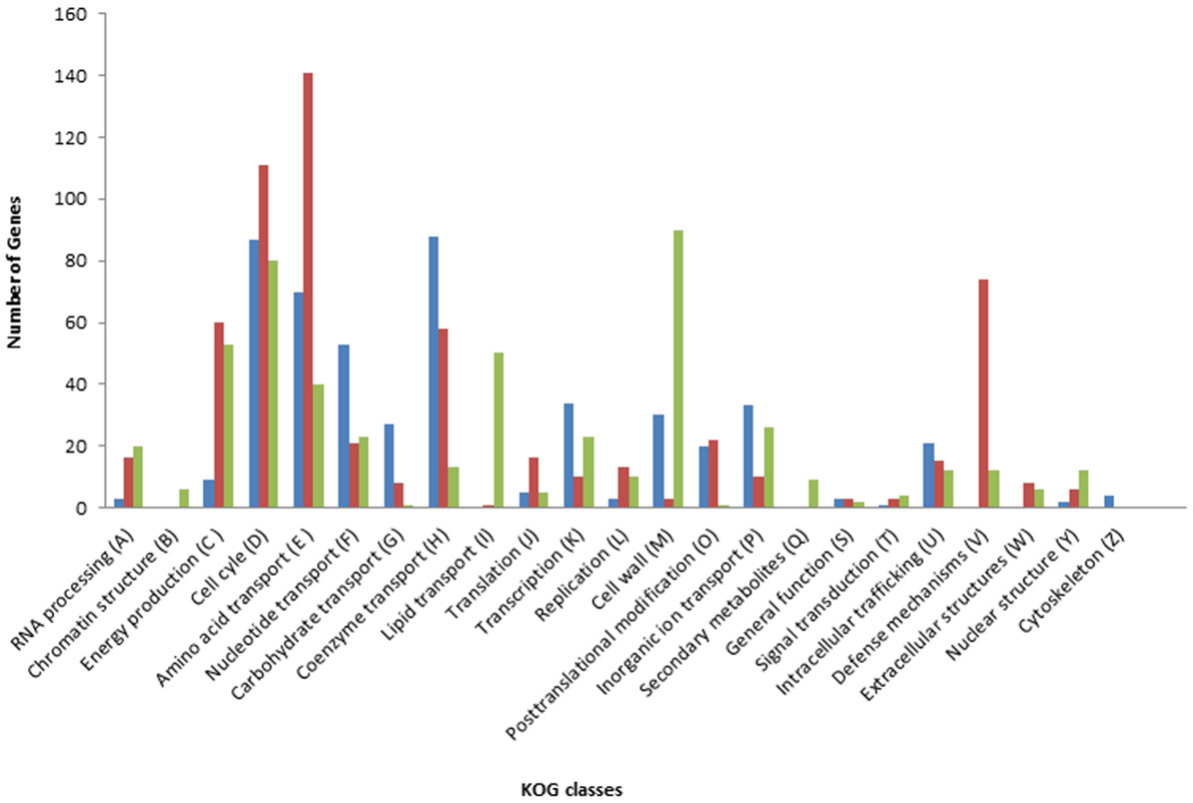
Functional classification of significantly enriched genes in mock- and rust-inoculated (0, 12 and 84 hai) leaves of common bean. The GO enriched genes are functionally classified based on EuKaryotic Orthologous Groups (KOGs). KOG categories are presented on X-axis and number of genes in the corresponding classes are shown on Y-axis.

GO enrichment analysis was carried out by using Panther [52] and functionally classified based on KOGs. In total, over 1000 reliable genes or transcripts were identified after considering enrichment score (>1.3), P-Value (<1.0E-1) and false discovery rate (<0.05). Of these, 451 (41.30%), 361 (33.06%) and 280 (25.64%) genes or transcripts belong to biological processes, cellular component and molecular function categories, respectively. The genes in the larger group, biological processes (41.30%) shared high homology with the genes involved in abiotic and biotic stress, defense response and signal transduction while those in the remaining groups (58.7%) shared the genes with functions related to cell structure, protein metabolism, transport processes, and transcription regulation (Fig 4). The observed enrichment score was highest for the stress related genes and R genes and were mostly represented at 12 hai, suggesting that the pathogen responsive signaling cascades were active in early inoculation.

### Transcription factors actively expressed in common bean-rust interaction

Earlier reports suggested that several transcription factors modulate the gene expression in biotic and abiotic stresses [53]. This study identified several genes and TFs that were actively expressed both in early and late defense responses in plant-fungal interactions. Among these, mainly six TFs, homeo-domain like, WRKY, Basic Leucine Zipper Domain (bZIP), myeloblastosis (MYB), No apical meristem; *Arabidopsis* transcription activation factor; and Cup-shaped cotyledon (NAC), and Centromere Binding Factor (CBF-1) (Table 7; S3 Table), and six genes including mitogen activated protein (MAP) Kinases, calcium dependent protein kinases, glutathione-S-transferases, calmodulin binding, pathogenesis like, glycosyl/hydrolase family proteins were differentially expressed during bean-rust inoculation. Also, the transcripts were compared against a legume transcription factor database (TFDB) to identify ten abundant TF families homeobox (HB), WRKY, bZIP, MYB, NAC, C3H-transcription factor 33 (C3H3), activating protein 2 (AP2), basic helix-loop-helix (bHLH), and plant homeodomain (PHD). Of these, WRKY, bZIP, MYB family of TFs were further confirmed by RT-PCR (discussed elsewhere in the text). In *Arabidopsis*, TF-DREB (dehydration responsive element-binding) has been reported in diverse abiotic stresses including drought, cold and high salt [54]. Also, plants have developed diverse defense mechanisms for scavenging abiotic and biotic stress inducing molecules by employing detoxifying enzymes. Here we identified six differentially expressed detoxifying enzymes including cationic peroxidase 3, catalase 2, ascorbate peroxidase-1, and -3 proteins, heavy metal transport/detoxification superfamily, and glutathione-S-transferase (S4A Table). Of these, ascorbate peroxidase 1 and 3, and cationic peroxidase 3 were up-regulated at 84 hai and down-regulated at 12 hai. On the other hand, catalase 2, and heavy metal transport/detoxification superfamily and glutathione-S-transferase genes were up-regulated at 12 hai when compared to 84 hai. Also, cytochrome P450 associated genes (16) that contain recognition sites for MYB and Myelocytomatosis (MYC) TFs that play an important role in plant defense mechanisms were highly expressed at 84 hai (S4B Table). Additionally, 11 chloroplast specific genes that have proposed roles in cellular metabolism and stress responses were identified (S4C Table). Within the chloroplast specific genes, the maximum difference in expression was observed between 84 hai and 12 hai. The stress hormones, abscisic acid (ABA) and salicylic acid (SA) induces tocophenol cyclase and we identified increased expression of tocophenol cyclase at 84 hai.

**Table 7:** Representative transcription factors (TFs) expressed in bean-rust interaction.

Gene ID	Log_2_FC			Description
	12Ivs0I	84Ivs0I	12Ivs84I	
**Phvul.001G001000**	2.91	-1.34	-4.25	Catalase 2; C2H2-type Zinc Finger Family Protein
**Phvul.001G025200**	1.21	-1.84	-3.05	Myb Domain Protein 73; Arginine Methyltransferase 11
**Phvul.001G028800**	-2.57	-0.19	2.38	BSD Domain-Containing Protein
**Phvul.001G056900**	-1.94	1.60	3.55	Protein of Unknown Function (DUF581); C3HC Zinc Finger-like
**Phvul.001G088200**	3.76	1.48	-2.28	WRKY DNA-Binding Protein 75; Beta Carbonic Anhydrase 5
**Phvul.001G100200**	2.09	-1.02	-3.11	NAC Domain Containing Protein 47; Basal Transcription Factor Complex Subunit-Related
**Phvul.001G124800**	-1.34	1.11	2.45	S-adenosyl-L-Methionine-Dependent Methyltransferases Superfamily Protein; Alfin-like 3
**Phvul.001G135500**	-0.85	1.54	2.39	Remorin Family Protein; C2H2 Zinc-Finger Protein SERRATE (SE)
**Phvul.001G141000**	-2.63	-1.17	1.46	Squamosa Promoter binding Protein-like 9
**Phvul.001G143800**	1.20	2.93	1.73	Aha1 Domain-Containing Protein; ARIA-Interacting Double AP2 Domain Protein
**Phvul.001G154700**	3.85	4.98	1.13	Heat Shock transcription factor A6B
**Phvul.001G211700**	N/A	4.32	N/A	myb Domain Protein 12; Cryptochrome 1
**Phvul.001G213600**	1.52	-2.34	-3.87	WRKY DNA-Binding Protein 69; ATP Binding Cassette Subfamily B19
**Phvul.001G224600**	-2.87	-0.62	2.25	Basic Helix-Loop-Helix (bHLH) DNA-Binding Superfamily Protein; Acyl-CoA N-Acyltransferase with RING/FYVE/PHD-type Zinc Finger Domain
**Phvul.001G226500**	-2.18	1.56	3.74	GATA Transcription Factor 16; Actin Binding Protein Family
**Phvul.002G012200**	1.67	-1.64	-3.30	LOB Domain-Containing Protein 38
**Phvul.002G012200**	1.67	-1.64	-3.30	LOB Domain-Containing Protein 38; Ankyrin Repeat Family Protein/BTB/PoZ Domain-Containing Protein
**Phvul.002G017600**	-0.10	-2.69	-2.59	Basic Helix-Loop-Helix (bHLH) DNA-Binding Superfamily Protein; Chitin Elicitor Receptor Kinase 1
**Phvul.002G056300**	2.21	2.08	-1.15	Homeobox-Leucine Zipper Protein 4 (HB-4)/HD-ZIP Protein
**Phvul.002G062000**	-0.70	3.08	3.78	Lactate/Malate Dehydrogenase Family Protein; Alfin-like 6

In pathogen treated samples, Pathogen Associated Molecular Patterns (PAMP) mediated regulation is the most common HR response elicited by the plant involving several gene regulatory cascades. The ABA-stress response (Asr) genes associated with ABA signaling pathway that play an important role in drought stress has been reported in common bean [55] and the role of ABA in biotic stress is becoming increasingly evident [56, 57]. The other phyto-hormones, salicylic acid (SA), jasmonic acid (JA) and ethylene (ET) hormones also play an important role in plant defense responses [58]. Plants combat necrotrophic and biotrophic pathogens by triggering JA, and SA-mediated signaling pathways. The plant growth regulator, ABA and the WRKY family of TFs act as molecular switches between the SA, and JA-dependent defense responses in plants against herbivores and necrotrophic pathogens [59]. The cross talk between signaling pathways in plants is evident in biotic and abiotic stresses. The presence of high levels of WRKY TFs and some SA signaling genes in the transcriptome data suggest the possible interaction in bean-rust resistance and will be explored in the future. Pathogen triggered immunity constitutes the first level of plant defense and restricts the pathogen from proliferation. Ten differentially expressed chitinase genes including chitinase-like protein and chitinase A that participate in PTI were identified in common bean (S4D Table). Compared to the expression of other PTI related genes, the expression of R genes including CC-NBS-LRR resistance protein and NBS-type resistance protein were significantly high. Also the heat shock protein 90.1 and NB-ARC domain containing disease resistance protein (RPM1) were highly expressed at 84 hai. In *A*. *thaliana*, RPM1 conferred resistance to *P*. *syringae* expressing either avrRpm, which causes hyperphosphorylation of the RPM1 interacting protein 4 (RIN4), which subsequently triggers disease resistance [19].

**Fig 5:**
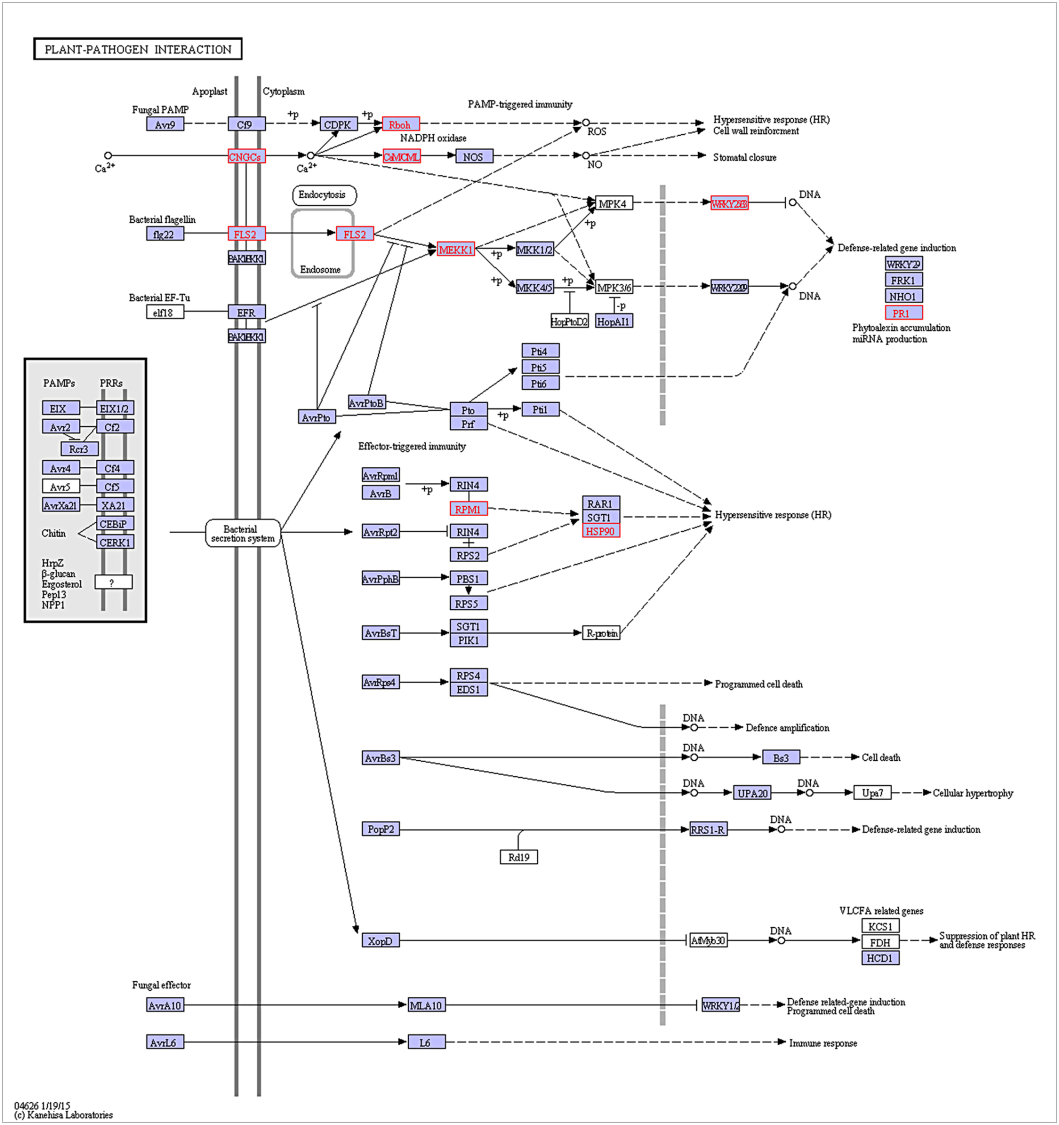
Putative KEGG pathway (ID: 04626) identified in plant-pathogen (common bean-*Uromyces appendiculatus*) interaction. The boxes highlighted in red are enzymes (10) identified in this study.

**Fig 6:**
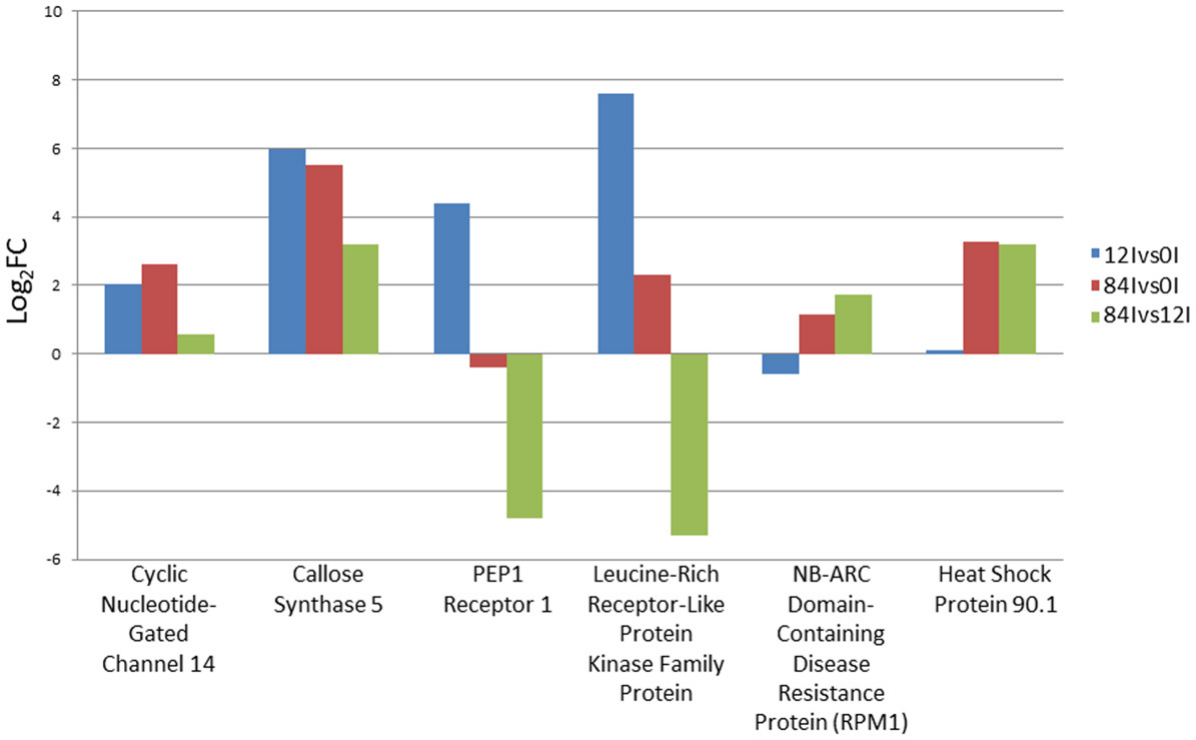
Differentially expressed genes identified in bean-bean rust interaction pathway (ID: 04626) at 0, 12 and 84 hai. The differential expression (Log_2_FC>2) of six selected genes (cyclic nucleotide-gated channel 14, callose synthase 5, PEP1 receptor 1, leucine-rich receptor-like protein kinase family protein, NB-ARC domain-containing disease resistance protein, and heat shock protein 90.1) at different time points is shown in the Figure.

The KEGG pathway analysis of differentially expressed transcripts in response to *U*. *appendiculatus* stress in common bean identified 324 pathways associated with physiological processes (S5 Table). Further, the functional classification revealed four major categories with number of genes include: i) metabolic pathways (704), ii) secondary metabolite biosynthesis (321), iii) microbial metabolism in diverse environments (117) and iv) ribosome structure (108). More specifically, we found one pathway that incorporates ten genes potentially involved in the bean-bean rust resistance response (Fig 5). For instance, three genes callose synthase 5 (CalS5), PEP1 receptor 1, and leucine-rich receptor-like protein kinase superfamily protein were abundantly expressed (fold change >2) in response to the bean-pathogen interaction (Fig 6; S6 Table). The putative functional roles of these proteins are discussed below. Callose Synthase 5 is an essential component of specialized cell walls such as callose wall, callose plugs and pollen tube wall, and its synthesis is induced by pathogen invasion, wounding and in response to environmental cues. PEP 1 Receptor 1 (PR1), a key component in PEPR pathway mediates defense responses following microbial recognition. In plants, PR1 triggers pathogen-induced systemic immunity. Zinc finger protein (ZPR1) present in the cytoplasm interacts with the receptor tyrosine kinase that has a proposed role in signaling while LRR motifs primarily aid in defending the host plant against pathogen invasion.

### Differences in histone modifications and transcription in response to rust interaction

The correlation between histone methylation and transcriptional inactivation was dissected by comparing the RNA-Seq and ChIP-Seq datasets to identify 265, 191, and 200 commonly expressed genes between 12Ivs0I, 84Ivs0I and 84Ivs12I, respectively (S7 Table). Among these, chr07 followed by chr11 and chr10 had maximum number of genes that overlapped in 12Ivs0I between RNA-Seq and ChIP-Seq datasets. Chr06 had the least number of genes as observed in H3K9_me2_ modification. Similarly, using a histone acetylation mark we identified 300 (12Ivs0I), 244 (84Ivs0I), and 249 (84Ivs12I) enriched genes/*cis*-elements that were in common between RNA-Seq and ChIP-Seq analysis. Of these, chr11 had maximum number of genes across the time points.

The methylation specific proteins that were concurrently found between RNA-Seq and ChIP-Seq include histone mono-ubiquitination 1 and histone H3K4-specific methyltransferase (SET7/9 family) and these proteins showed high level of expression at the time point 12 hai. Similarly, the acetylation modifications commonly identified between acetylation and transcriptome regulation include histone acetyltransferase of the cAMP Response Element Binding (CREB)-Binding Protein (CBP) family 12, histone deacetylase 3, and histone superfamily proteins (Table 8). Using the comparative approach, three highly expressed (Log_2_FC>2) defense responsive (low-molecular-weight cysteine-rich 68, gigantea protein and chaperone DnaJ-domain superfamily proteins) and three R proteins (pleiotropic drug resistance protein 12, MATE efflux family and NB-ARC domain containing) were identified concomitantly in transcriptome data, histone methylation, and acetylation modifications. In total, five different types of the LRR family of proteins, LRR, NB-ARC, CC-NBS-LRR, TIR-NBS-LRR and GTPase Ras group related LRR proteins that have possible roles in rust fungal interaction in common bean were identified. Three resistant proteins, pleiotropic drug responsive gene 12, LRR, and multidrug resistance associated protein 9 were uniquely identified between 12 hai, supporting the role of R proteins in HR-signaling. The pleiotropic drug resistance protein 12 was concurrently enriched with histone methylation and acetylation marks and in transcriptome data.

**Table 8:** Differentially marked H3K9_me2_ and H4K12_ac_ modifications and transcription in common bean-rust interaction.

Gene Id	Description	RNA-Seq			ChIP-Seq-H3			ChIP-Seq-H4		
		12Ivs0I	84Ivs0I	84Ivs12I	12Ivs0I	84Ivs0I	84Ivs12I	12Ivs0I	84Ivs0I	84Ivs12I
**Phvul.008G141100**	Histone Mono-ubiquitination 1	0.67	0.52	-0.15	1.31	-	0.93	-	-	-
**Phvul.008G138500**	Histone H3 K4-Specific Methyltransferase SET7/9 Family Protein	0.08	0.05	-0.02	0.62	0.73	-	-	-	-
**Phvul.001G186300**	Histone Deacetylase 3	0.39	0.04	0.43	-	-	-	0.6	0.68	1.22
**Phvul.010G165900**	Histone Acetyltransferase of the CBP Family 12	1.49	0.24	-1.24	0.75	0.77	-	-	1.53	1.59
**Phvul.006G057800**	Histone Superfamily protein	-	-	-		-	-	0.82	0.71	-

In HR-signaling cascades, it has been suggested that the pathogenesis factor interacts with R proteins that act as first line of defense molecules. In plants, the activation of R genes triggers ion flux and ultimately results in oxidative burst (reactive oxygen species, ROS) of the affected cells. Other than plant resistant proteins, the highly expressed members were: peroxidase superfamily proteins, NAD(P)-binding Rossmann-fold superfamily and FAD/NAD(P)-binding oxidoreductase family proteins. Also, two stress responsive proteins, beta-fructofuranosidase 5 and protein kinase superfamily were uniquely expressed at 12 hai. The other proteins significantly enriched in both the datasets and their respective functions were provided (Table 9). Among these, two carbohydrate metabolism associated proteins including ribulose-bisphosphate carboxylase and ATP synthase delta/epsilon chain were highly expressed at 12 hai than at 84 hai.

**Table 9:** Genes and their functions associated with histone modifications and transcription in common bean-rust interaction.

Gene Id	Description	Function
**Phvul.008G214400**	Transducin/WD40 Repeat-like Superfamily Protein	RING finger, transcriptional corepressor
**Phvul.005G061100**	Ribulose-Bisphosphate Carboxylases	Carbon fixation
**Phvul.005G173700**	Pleiotropic Drug Resistance 12	ABC transporter
**Phvul.005G174000**	Nucleotide Transporter 1	TLC ATP/ADP transporter
**Phvul.008G071300**	NB-ARC Domain-Containing Disease Resistance Protein	Resistant protein
**Phvul.010G094800**	NAD(P)-Binding Rossmann-Fold Superfamily Protein	Oxidoreductase activity
**Phvul.001G103300**	MATE Efflux Family Protein	Multi-drug resistant protein
**Phvul.005G071400**	Low-Molecular-Weight Cysteine-rich 68	Defense response
**Phvul.007G135900**	Integrase-type DNA-Binding Superfamily Protein	Regulation of transcription
**Phvul.004G088300**	Gigantea Protein (GI)	Stress response
**Phvul.007G017100**	FAD/NAD(P)-binding Oxidoreductase Family Protein	Carotenoid biosynthetic process
**Phvul.001G226300**	Chaperone DnaJ-Domain Superfamily Protein	HSP, stress response
**Phvul.001G036700**	Beta-Fructofuranosidase 5	Glycosyl hydrolases family
**Phvul.010G031400**	ATP Synthase Epsilon Chain	ATP synthesis coupled proton transport
**Phvul.010G078600**	Alanine:glyoxylate Aminotransferase 3	Aminotransferase class-III, transaminase activity

### ChIP-qPCR analysis in common bean-rust interaction

**Fig 7:**
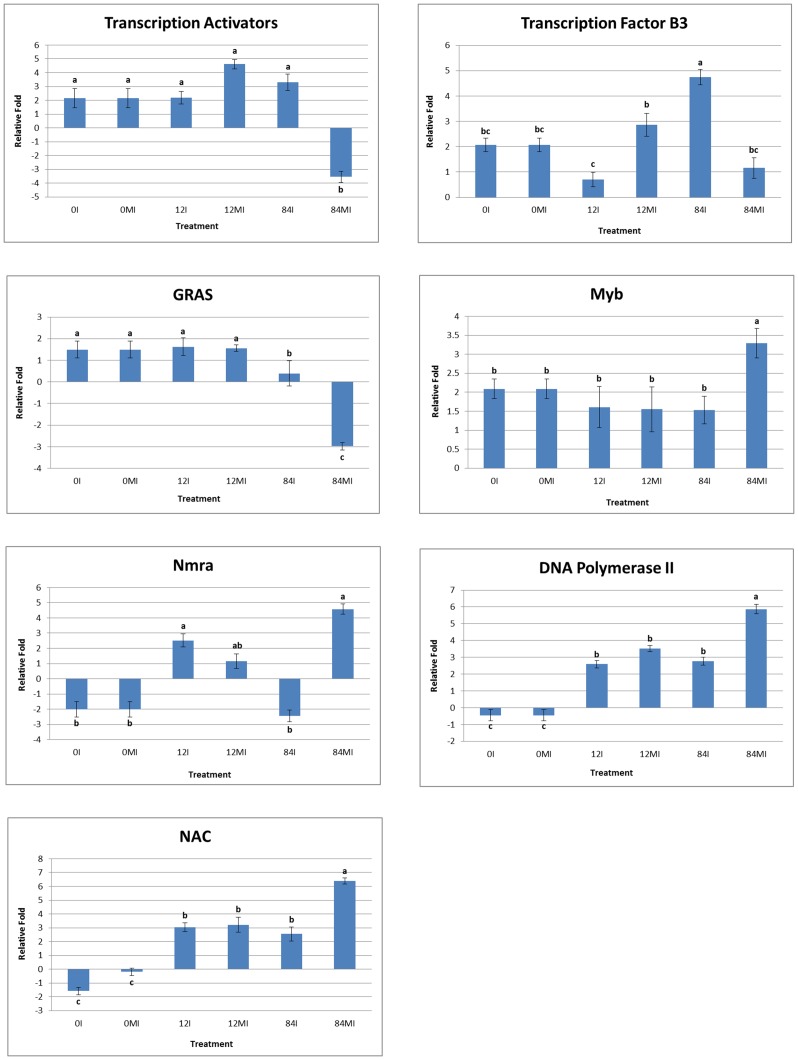
Validation of the relative expression levels of seven selected genes from ChIP-Seq analysis by quantitative Real-Time RT-PCR (qPCR). Expression pattern of selected common bean genes in mock-inoculated and rust-inoculated leaf samples at different time points. The x-axis shows different time point and y-axis shows relative fold change value (Log_2_FC). Different alphabets in this Figure indicate statistically significant (p-value<0.05) difference in relative expression of each gene between different time points (0, 12, 84 hai) for both inoculated and mock-inoculated leaf samples. The qPCR results from two technical replicates were subjected to ANOVA by using Minitab17 software.

We also analyzed immunoprecipitated DNA for acetylation (H4K12_ac_) and methylation (H3K9_me2_) modifications using real-time PCR to validate the differences in binding between H3 and H4 at 0, 12 and 84 hai. The list of primers used for real-time PCR is given in S8A Table. Our ChIPed real-time PCR results overlapped with the ChIP-Seq analysis. At 0 hai, transcriptional activator, transcription factor B3 and Gibberellic Acid Insensitive (GAI)-Repressor of Ga1 (RGA)-Scarecrow (SCR) (GRAS) family of transcription factors were associated with the activation mark H4K12_ac_ while NAC-transcriptional gene factor-like 9, DNA Pol II transcription factor and Nmra-like negative transcriptional regulator family of genes were marked by H3K9_me2_. The importance of transcription factors in mediating the histone modifications in biotic stresses is increasingly evident [60]. The marking of transcriptional activator and transcription factor B3 family protein was higher at 84 hai than 12 hai post-inoculation. However, GRAS family transcription factor showed higher expression at 12 hai when compared to 84 hai (Fig 7). Similar to our study, GRAS TFs were seen to accumulate in the defense response to *P*. *syringae* pv. in tomato [19]. Similarly, the role of heat shock transcription factor B3 (HSFB3) has been implicated in response to abiotic stress and in mycorrhyzal association [61]. We observed a significant change in HSFB3 marking between 12 and 84 hai. In soybean, the NAC transcription factor was seen to regulate 72 genes that were active in seedling development [62]. The NAC-domain containing TF was upregulated by 4-fold at 12 hai with the methylation (H3K9_me2_) mark. Only a negligible change was observed in expression between 12 hai and 84 hai inoculation for Myb-like HTH transcriptional regulator family protein with H4K12_ac_ mark and Nmra-like negative transcriptional regulator family protein and DNA Pol II transcription factors with H3K9_me2_ mark. The results showed that all seven genes from ChIP-Seq were significantly (p-value<0.05) expressed between 0, 12 and 84 hai.

### RT-PCR and real-time RT-PCR validation of RNA-Seq analysis

Due to increased sensitivity and high throughput, RNA-Seq and ChIP-Seq have become the choice of deep sequencing technologies for comprehensive gene expression and regulation studies in several plant species. The comparative RNA-Seq and ChIP-Seq analysis revealed 501 differentially expressed genes that were common in both data sets. Among these, seven genes were selected to confirm their expression level by utilizing reverse transcriptase PCR (RT-PCR) and real-time quantitative RT-PCR (qPCR). In order to confirm that RNA used in these experiments was not contaminated with DNA, we amplified gDNA and cDNA with SB1, a common bean specific molecular marker [27]. Primers derived from the SB1 sequence were used to amplify a 420 bp product from genomic DNA and cDNA. As expected SB1 amplified in genomic DNA, but not in cDNA (S2A Fig). Interestingly, primers from NAC-transcriptional gene factor-like 9 (Phvul.010G120700) amplified long flanking intronic genomic DNA yielding a 963 bp amplicon and a 731 bp amplicon in cDNA (S2B Fig).

**Fig 8:**
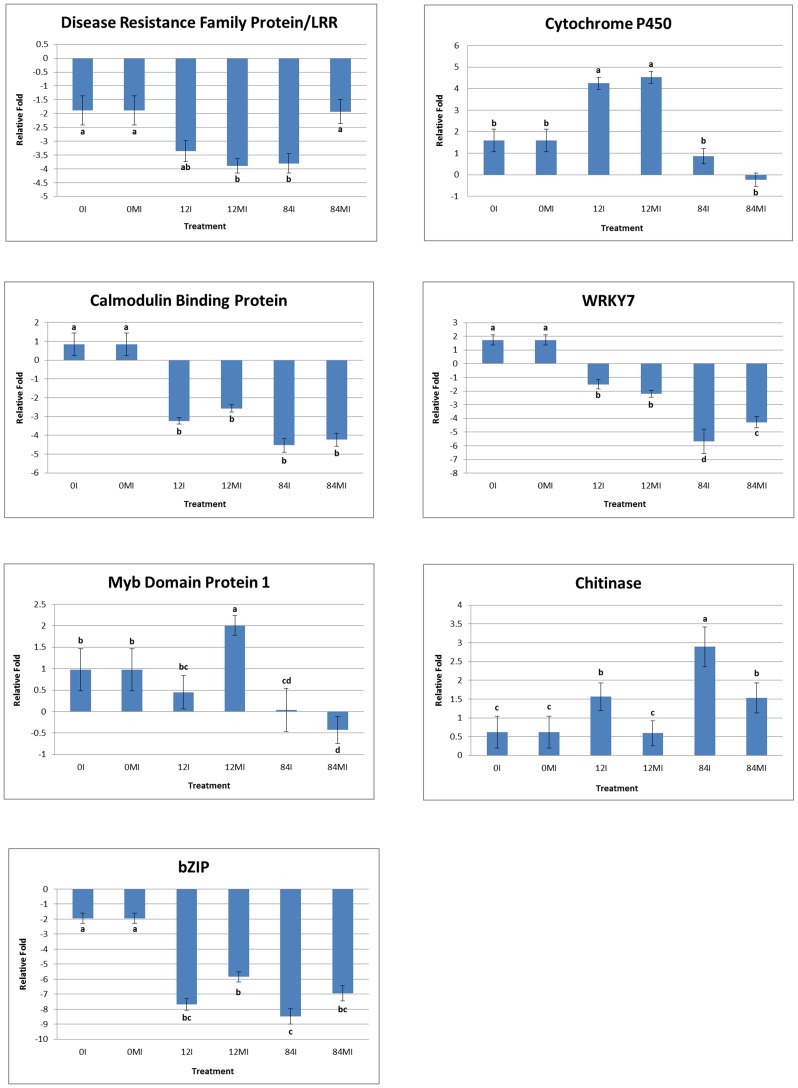
Validation of the relative expression levels of seven selected genes from RNA-Seq analysis by quantitative Real-Time RT-PCR (qPCR). Expression pattern of selected common bean genes in mock-inoculated and rust-inoculated leaf samples at different time points. The x-axis shows different time point and y-axis shows relative fold change value (Log_2_FC). Different alphabets in this Figure indicate statistically significant (p-value<0.05) difference in relative expression of each gene between different time points (0, 12, 84 hai) for both inoculated and mock-inoculated leaf samples. The qPCR results from two technical replicates were subjected to ANOVA by using Minitab17 software.

To qualitatively and quantitatively validate differentially expressed transcripts from RNA-Seq, RT-PCR and qPCR were performed across three different time points (0, 12, and 84 hai) on seven defense responsive genes. These genes were selected because they were differentially expressed in common bean based on our RNA-Seq analysis, and also as they played an important role in defense responses of other legumes against the fungal pathogen, *Cercosporidium personatum* in peanut and bacterial pathogen *Xanthomonas axonopodis* in soybean [63, 64]. The primers were designed for selected genes using GenScript real-time PCR (TaqMan) primer design tool; the list of primers is given in S8B Table. The genes included encode LRR family, cytochrome P450, calmodulin binding, chitinase, WRKY, MYB, and bZIP families of TFs. The LRR family, calmodulin-binding and MYB like TFs as identified here have been reported in response to *Botryosphaeria dothidea* infection in poplar [65]. Different classes of LRR proteins that play an important role in plant immune responses have been reported. They act as the first line of defense by mediating response through SA signaling pathway, as reported in resistance to *P*. *syringae* proteins in *Arabidopsis* [19]. Recently, the role of NBS-LRR family proteins in defense responses has been reported in Jatropha and castor bean [66]. In our study, we observed a two-fold decrease in expression of LRR family of proteins in response to *U*. *appendiculatus* in common bean between 0 hai to 84 hai. A number of wound-responsive genes including cell wall modifying enzymes, signaling molecules and secondary metabolites were active in plant defense responses while the pathogen is invading the host [67]. Of these, some osmotic stress-related and heat shock-regulated genes such as chitinase, calmodulin and bZIP transcriptions were identified in this study. Chitinases have been previously reported in plant-pathogen interactions as a defense response [68]. In the present study, a positive correlation between chitinase activity and common bean-rust resistance was found. Real-Time quantitative PCR (qPCR) results showed higher levels of chitinase in inoculated samples than mock-inoculated (Fig 8). Our results concurred with the previous study that showed increase in chitinase activity in response to *B*. *dothidea* infection in poplar, confirming the antifungal properties in plants [69]. *BZIP* proteins are large group of TFs that are conserved across eukaryotes including monocots and dicots, which are structurally characterized by the presence of i) a basic region that binds to DNA and ii) a leucine zipper that is involved in protein homo- and hetero-dimerization [70]. *BZIP* gene families have been previously reported in response to pathogen attack in different plant species including *Arabidopsis*, rice, maize, sorghum, castor bean, cotton and poplar [71]. Similarly, in common bean bZIP transcription factors were up-regulated in response to *U*. *appendiculatus* pathogen. The RT-PCR results revealed that a large difference in expression was observed for bZIP transcription factors family of proteins.

In the common bean-rust interaction, we anticipated possible cross-talk between stress related genes and TFs. For instance, elevated calmodulin levels have been reported in response to avirulent pathogens or flagellin or salicylic acid in *Arabidopsis* [72]. Independently, the cross-talk between calmodulin and WRKY-regulated gene networks has been proposed in *P*. *syringae* infection in *Arabidopsis* [73]. In our study, the calmodulin levels were elevated at 12 hai when compared to 84 hai, supporting the idea of early HR responses in Sierra to bean-rust. In *Arabidopsis*, the inoculation of *Alternaria brassicicola* and *A*. *alternata* increased transcript levels of cytochrome p450 family of genes by ten-fold [74]. In this study, cytochrome p450 was up-regulated across the time points, but a three-fold increase was observed at 12 hai, suggesting its role in early defense response than in later interaction. Supporting the idea of cross-talk in disease R signaling, the upstream sequences of cytochrome p450 genes contain recognition sites for MYB, MYC and WRKY transcription factors that modulate plant defense responses. The relative expression levels of these *cis*-acting elements were compared.

The members of the MYB transcription factor family have been implicated in flavonoid biosynthesis and in defense responses [75]. Several variants in MYB genes have been identified and characterized in plant-pathogen interactions. In our study, MYB transcription factors were highly expressed during early time points and under-expressed in late time points. WRKY is a large family of transcription factors that have been reported in abiotic and biotic stresses. Previously, homologs of WRKY transcription factors have been identified in defense responses against *Phytophthora spp*. in potato and *Xanthomonas spp*. in rice [76]. In this study, we identified differentially expressed WRKY transcription factors that have implicated roles in downstream regulation during *U*. *appendiculatus* interaction in common bean. For the majority of the genes selected, the qRT-PCR expression profiles were in concurrence with the RNA-Seq data. The results showed that all seven genes from ChIP-Seq were significantly (p-value<0.05) expressed between 0, 12 and 84 hai. To corroborate our real-time PCR results, further were re-validated the seven genes by reverse-transcriptase PCR, where *cons7* was used as a control (S3 Fig). In the seven genes analyzed, consistently, 12 hai showed higher levels of expression when compared to 84 hai, supporting the role of defense responsive genes in early plant-pathogen interactions.

## Materials and Methods

### Plant material and growth conditions

Common bean cultivar “Sierra” was used for all experiments in this study. Sierra was derived from a crossing of Mesoamerican commercial pinto varieties with navy and black bean breeding lines at Michigan State University in the early 1980s. Selections of progeny were made over a period of nine seasons for traits such as seed size, color, and rust resistance [77]. Three days after seed germination on moist filter paper, seedlings were transferred to plastic pots as previously reported [27], and maintained in the green house at 28/20°C and 14/10 h photoperiod (Delaware State University, Dover, Delaware). Leaves from 10 two-week-old plants were inoculated with fungal rust *Uromyces appendiculatus* (race 53). In total, 90 plants were used in this study from which samples collected at three different time points after inoculation (0, 12, and 84 hai), two treatment conditions (inoculated and mock-inoculated) and three biological replicates (R1, R2 and R3). For each time point, 30 plants were maintained with 10 plants per replicate, among which five plants were inoculated with rust pathogen while five were mock-inoculated. Mock-inoculated plants were sprayed with water containing 0.01% of Tween 20 and served as controls. Plants from the naturally rust-susceptible cultivar “Olathe” served as a control to confirm the success of inoculation. Leaves were collected from both inoculated and mock-inoculated plants at 0, 12 and 84 hours-after-inoculation (hai) for ChIP-Seq and RNA-Seq experiments. All samples were flash frozen with liquid nitrogen and stored at -80°C.

### Isolation and immunoprecipitation of chromatin

ChIP assay was performed as described previously by the Lam Laboratory [33], and modified for common bean. Briefly, samples were ground to a fine powder in liquid N_2_ and fixed in cold nuclear isolation buffer containing 1% formaldehyde with 20 μl of protease inhibitor (#87786, Thermo Scientific, Rockford, IL) at room temperature. The cross-linking reaction was terminated with 2 M glycine. The lysate was filtered through one layer of Mira cloth (Calbiochem, SanDiego, CA) into a centrifuge tube and nuclei were pelleted at 3000 g for 10 min at 4°C. The pellet was vortexed and re-suspended in 300 μl of cold nuclear isolation buffer without formaldehyde. The nuclear suspension was transferred to equal volume of 15% Percoll solution, centrifuged at 3000 g for 5 min and resuspended in nuclei lysis buffer. The nuclear lysate was sonicated five times, each for a 15 s pulse on power 6 using a Soniprep 150 (MSE (UK) Ltd, London, UK), to shear DNA to approximately 100–350 bp fragments (S4 Fig). The nuclear lysate was diluted 10 times with ChIP dilution buffer. 100 μl sample of chromatin was incubated with 5 μl of rabbit polyclonal antibody that was raised against a synthetic peptide corresponding to the N-terminus of histone H4 acetylated at K12 (#A-4029-050; Epigentek, Farmingdale, NY). Similarly, 5 μl of mouse epitope region of histone H3 di-methylated at amino acid from 1–18 (#17–681; Millipore, San Diego, CA) antibody was used for incubation with chromatin in 900 μl of ChIP dilution buffer at 4°C overnight. This study utilized two histone modifications, H3K9_me2_ and H4K12_ac_. In *Arabidopsis*, the H3K9_me2_ mark has been evaluated at the whole-genome level using chromatin immunoprecipitation (ChIP) followed by tiling microarray analysis, ChIP-chip [78]. Also, they identified preferential localization of H3K9 methylation in heterochromatin. On the other hand, chromatin marked by H4K12_ac_ is primarily localized in the active coding regions of the genome and therefore facilitates binding of transcriptional factors to promote transcription [79]. Hence, we selected these two histone marks to understand the genome-wide changes in active and inactive sates of chromatin in rust-inoculated and mock-inoculated common bean cultivar Sierra. Twenty μl of Pierce protein A/G magnetic beads (#88802, Thermo Scientific, Rockford, IL) was added to the sample and incubated for 3 h at 4°C followed by incubation with Goat-anti-rabbit IgG antibody (#ab72465; Cambridge, MA). The magnetic beads were collected by centrifugation and washed three times with ChIP dilution buffer. The antibody-chromatin complex was washed, eluted, and de-cross-linked with 20 μl of 5 M NaCl at 65°C for overnight. The samples were digested with Proteinase-K (Thermo Scientific, Rockford, IL at 45°C for 2 h. DNA was obtained by phenol-chloroform extraction and subsequent ethanol precipitation was resuspended in 20 μl of TE buffer. For each treatment 100 ng of purified DNA was used to generate the ChIP-Seq library using Illumina HiSeq 2500 (Illumina Inc., San Diego, CA), which was sequenced at the Delaware Biotechnology Institute (Newark, DE). The mock-inoculated samples for each of the time points in the three biological replicates served as a control for the inoculated samples during bioinformatic analyses.

### RNA isolation

Total RNA was extracted from inoculated and mock-inoculated frozen leaf samples collected at 0, 12 and 84 hai using TRIzol (#15596–026, Ambion, Carlsbad, CA) according to the manufacturer’s protocol. Residual genomic DNA in RNA was removed by DNase I treatment as per the instruction (#AM1906, Ambion, Carlsbad, CA). In all RNA samples, reagent contamination (A260/A230 nm ratios) and protein contamination (A260/A280 nm ratios) were determined by Nanodrop 2000 spectrophotometer (Thermo Scientific, Wilmington, DE) and only the samples with OD260/280 >1.8 were utilized for sequencing and downstream validation. The RNA purity/quality was assessed by agarose gel electrophoresis (1.2%) and Bioanalyzer 2100 (Agilent Technologies, Santa Clara, CA) based on 28S/18S rRNA band intensity (2:1) and RNA integrity number (RIN) >8, respectively. The quantity of high quality RNA (RIN>8) ranged between 0.8–1.0 μg in all samples.

### Library construction and sequencing

The quality, purity and size of the immunoprecipitated DNA samples were determined by AATI Fragment Analyzer (Ames, IA). The ChIP-Seq libraries (50 bp) were prepared by using Illumina TruSeq ChIP Sample Preparation kit (#IP-202-1012; Illumina Inc., San Diego, CA) as per the manufacturer’s instruction. Similarly, the RNA quality and purity were assessed using Fragment Analyzer (Ames, IA). RNA-Seq libraries (50 bp) were prepared by utilizing Illumina TruSeq Stranded mRNA Sample Preparation kit (#RS-122-2101; Illumina Inc., San Diego, CA) as per the guidelines. The high throughput data generated in this study is submitted to the SRA section of the NCBI with the bioproject number PRJNA280864 for ChIP-Seq and RNA-Seq experiments.

### ChIP-Seq data analysis workflow

Base calling was performed in real-time from the sequence signals and demultiplexed FASTQ files were generated using Consensus Assessment of Sequence And VAriation (CASAVA). Raw reads were collected and their quality was assessed using FastQC to determine data statistics such as number of reads, individual nucleotide count, total number of nucleotides, and GC percentage. Raw reads were then trimmed and filtered to remove low quality data, and mapped to the *Phaseolus vulgaris* G19833 genome (Phytozome version 1.0) with no more than two mismatches by using Bowtie v1.0 [80]. The methylation (H3K9_me2_) and acetylation (H4K12_ac_) marked peaks were identified using Spatial clustering for Identification of ChIP-Enriched Regions (SICER) and annotated by using HOMER from both inoculated and mock-inoculated samples. A stern filtering was made while identifying differentially marked peaks. A gene was regarded as being methylated (H3K9_me2_-modified) or acetylated (H4K12_ac_-modified) only if it overlaps (based on ‘known’ annotated genes) with peak coordinates at least by one base.

### Transcriptome analysis

Sequence quality was evaluated by FastQC (v 0.10.1) and reads were trimmed for adapters and low-quality reads were filtered (Phred score < 30) by using FASTX toolkit (v 0.0.13) and resultant high-quality reads of at least 50 bases were retained (~97% of total reads). The quality reads thus collected were mapped against the reference genome using TopHat (v 2.0.9) with default parameters. The genome annotations (.gff3 file) available at Phytozome were used to extract features from transcriptome analysis. HTSeq (v 0.5.3p7) was used to generate raw read counts per gene from each sample using TopHat output and the known gene annotations. The resulting annotation information (.bam files) was used to determine differential gene expression using Cufflinks (v 2.0.2) suite of programs [81]. Cufflinks uses the option of multi read correction, which carries out an initial estimation procedure to more accurately account for the reads mapped to multiple locations in the genome by adding weights. Cuffdiff uses the weights thus generated to calculate Fragments Per Kilobase of exon per Million fragments mapped (FPKM) values and then differential gene expression is ascertained by pairwise comparisons between the datasets.

### Gene ontology and pathway analysis

Differential expression and gene enrichment analyses in mock-inoculated and inoculated leaf samples at different time points was carried out by using Cufflinks following functional annotation by PANTHER [82]. Further, we categorized the genes associated with the bean-rust interaction based on KOG functional classes. The hypergeometric test with multiple adjustments [83] was used for GO analysis and categorized into their respective classes or pathway annotations based on the Kyoto Encyclopedia of Genes and Genomes (KEGG; http://www.genome.jp/kegg/kegg2.html).

### RT-PCR and real-time quantitative RT-PCR validation

The Reverse Transcriptase-PCR (RT-PCR) and real-time quantitative RT-PCR (qPCR) validations were carried out to qualitatively detect the gene (mRNA) expression and to quantitatively measure the amplification of cDNA by using MyCycler thermocycler (Bio-Rad Laboratories, Hercules, CA) and ABI 7500 real-time PCR (Applied Biosystems, Foster City, CA), respectively. We selected seven defense responsive genes that are differentially expressed based on RNA-Seq analysis and seven genes differentially marked by H4K12_ac_ (4 genes) and H3K9_me2_ (3 genes) from ChIP-Seq analysis, representing active and inactive chromatin states in bean-rust interactions. Also, these genes have been previously reported as disease-resistance related in soybean [84] and *Arabidopsis* [85]. The details of genes and respective primers are given in the S8A and S8B Table. The primers for the selected defense responsive genes were designed by using the online tool for real-time PCR (TaqMan) primer design (GenScript USA Inc., Piscataway, NJ) and utilized for qualitative and quantitative determination of gene expression. The high quality RNA (RIN>8) of 0.8–1.0 μg derived from inoculated and mock-inoculated leaves and was reverse transcribed to first-strand complementary DNA (cDNA) with Oligo dT using Superscript III First Strand Synthesis System (Life Technologies, Carlsbad, CA) according to manufacturer’s instruction. RT-PCR was carried out under standard PCR conditions (94°C for 30 s, 60°C for 30 s, and 72°C for 30 s) for 30 cycles. The amplified products were separated in 2% agarose gel stained with Ethidium Bromide. Separately, real-time quantitative PCR (qPCR) was performed in 25 μl reactions that contained 10 ng of cDNA (or) immunoprecipitated DNA, 10 μM of primer pairs (FW and REV) and 12.5 μl of SYBR Green PCR Master Mix. PCR conditions for qPCR were as follows: 95°C for 10 min, followed by 40 cycles of 95°C for 15 s and 65°C for 1 min. In this study, we used three biological replicates, two treatments (mock-inoculated and inoculated), three collection time points after inoculation (samples collected at: 0, 12, 84 hai), and two technical replicates were maintained for both RT-PCR and qPCR analyses. To normalize the results, *cons7* was used as a gene of control for all tissue samples. The efficiency of primers was tested and analyzed by using previously reported 2-ΔΔCT method [86], where ΔΔCT = (CT of gene—CT of *cons7*) tissue to be observed—(CT of genex—CT of *cons7*) leaf tissue. The normalized CT values (ΔΔCT) from qPCR analysis were collected and analyzed by using Minitab 17, the expression results were presented as mean±SE. One-way ANOVA was performed on qPCR experiments for multiple comparisons between the mean of samples.

## Conclusions

This is the first comprehensive and integrated study that combines genome-wide profiling of histone modifications and gene expression in common bean, particularly under biotic stress. Collectively, this unified study identified 1,235 methylated, 556 acetylated, and 1,763 differentially expressed transcripts in the common bean-rust interaction respectively. The combined ChIP-Seq and RNA-Seq analysis identified defense responsive genes such as, calmodulin, cytochrome p450, chitinase, DNA Pol II and LRRs. Seven abundantly found transcription factor families across three time points (0, 12 and 84 hai) include WRKY, bZIP, MYB, HSFB3, GRAS, NAC and NMRA in common bean-rust interaction, which were further validated by real time PCR. The differential methylation and acetylation patterns observed here modulated the gene expression of defense related genes substantially. Among the significantly enriched genes, plant resistant (R) genes, detoxifying enzymes, and genes involved in physiological processes were predominant, supporting the idea of regulation of R genes and associated ion flux in HR responses. The presence of abundant R genes expressed at 12 hai compared to other time points, suggests that the early HR responses successfully elicited the downstream defense responses against the pathogen in a resistant cultivar such as Sierra. The putative pathways and key genes identified in this study provide a basis for further understanding the plant-pathogen interactions. In non-model species, the combined histone modifications and gene expression analysis is very limited and this study provides a comprehensive resource for epigenomic regulation in plants.

## Supporting Information

S1 Table ChIP-Seq analysis of differentially marked histone acetylation and methylation regions in bean-bean rust interaction at 0, 12 and 84 hai. (XLSX)

S2 Table RNA-Seq analysis of significantly enriched genes identified in bean-bean rust interaction at 0, 12 and 84 hai. (XLSX)

S3 Table Comprehensive list of transcription factors identified in bean-bean rust interaction. (XLSX)

S4 Table List of important genes identified from RNA-Seq Analysis. (A) List of significantly enriched detoxifying enzymes from RNA-Seq analysis (XLSX), (B) List of significantly enriched cytochrome p450 Genes from RNA-Seq analysis (XLSX), (C) List of significantly enriched-photosynthesis genes from RNA-Seq analysis (XLSX), (D) List of chitinase genes identified from RNA-Seq analysis.
(XLSX)

S5 Table Comprehensive list of KEGG pathway genes identified in bean-bean rust interaction. (XLSX)

S6 Table Top six differentially expressed genes identified in bean-bean rust interaction based on RNA-Seq analysis. (XLSX)

S7 Table List of genes identified from the combined analysis of ChIP-Seq and RNA-Seq in bean-bean rust interaction at 0, 12 and 84 hai. (XLSX)

S8 Table **(A) List of primers selected for real-time quantitative PCR (qPCR) from RNA-Seq**. **(B) List of primers selected for real-time quantitative PCR (qPCR) from ChIP-Seq**.
(XLSX)

S1 Fig Genes associated with epigenetic regulation. The percentage of genes associated with DNA-methylation, histone-methylation, histone-acetylation, chromatin remodeling, and polycomb group has been identified from the combined analysis of ChIP-Seq and RNA-Seq.
(TIF)

S2 Fig Quantification of DNA purity in reverse transcriptase PCR. (A) Common bean marker SB, linked to the *Ur-3* rust resistance locus amplify a 460 bp from genomic DNA but failed to amplify the cDNA (Lane 1: 100 bp ladder; Lane 2: SB1 gDNA; Lane 3: SB1 cDNA; Lane 4: Negative control-1 (no reverse transcriptase was added to cDNA synthesis); Lane 5: Negative control-2 (H_2_O only); Lane 6: 100 bp ladder). (B) Primers from NAC-transcriptional gene factor-like 9 (Phvul.010G120700) amplified intronic gDNA yielding a 963 bp amplicon from gDNA and 731 bp amplicon from cDNA. The order and contents of lanes 7 to 12 are identical to those in panel A.
(TIF)

S3 Fig Reverse transcriptase PCR of cDNA from seven selected transcripts identified from RNA-Seq. The Figure illustrates the products amplified by using RT-PCR from seven selected genes. Lane 1: 100 bp ladder; Lanes 2: gDNA of SB1; Lane 3: leaf cDNA, Lane 4: Negative control-1 (no reverse transcriptase added to SB1 cDNA), Lane 5: Negative control-2 (H_2_O), Lane 6: Positive control (*cons7*). Lanes 8–13: DREP/LRR, Lanes 14–19: Cytochrome p450, Lanes 22–27: Calmodulin, Lanes 28–33: WRKY-7 TF, Lanes 34–39: Myb like TF, Lanes 42–47: Chitinase, and Lanes 48–53: bZIP TF. Other lanes with 100 bp ladder: 7, 20, 21, 40, 41 and 54.
(TIF)

S4 Fig Common bean chromatin before and after the sonication. The chromatin was sonicated five times, each for a 15 s pulse on power 6 using a Soniprep, to shear DNA to approximately 100–350 bp fragments. (A) Chromatin from 12h rust-inoculated and mock-inoculated samples. Lane 1: 100 bp ladder; Lanes 2–3 mock-inoculated and inoculated samples before sonication; Lanes 4–5 mock-inoculated and inoculated samples after sonication. (B) Chromatin from 84h rust-inoculated and mock-inoculated samples. Lane 1: 100 bp ladder; Lanes 2–3 mock-inoculated and inoculated samples before sonication; Lanes 4–5 mock-inoculated and inoculated samples after sonication.
(TIF)
