# Supplementary figures and images for: Genome-Wide Profiling of Histone Modifications (H3K9me2 and H4K12ac) and Gene Expression in Rust (Uromyces appendiculatus) Inoculated Common Bean (Phaseolus vulgaris L.)
Source: PLoS One. 2015 Jul 13;10(7):e0132176. doi: 10.1371/journal.pone.0132176 (PMC4500563; doi:10.1371/journal.pone.0132176)

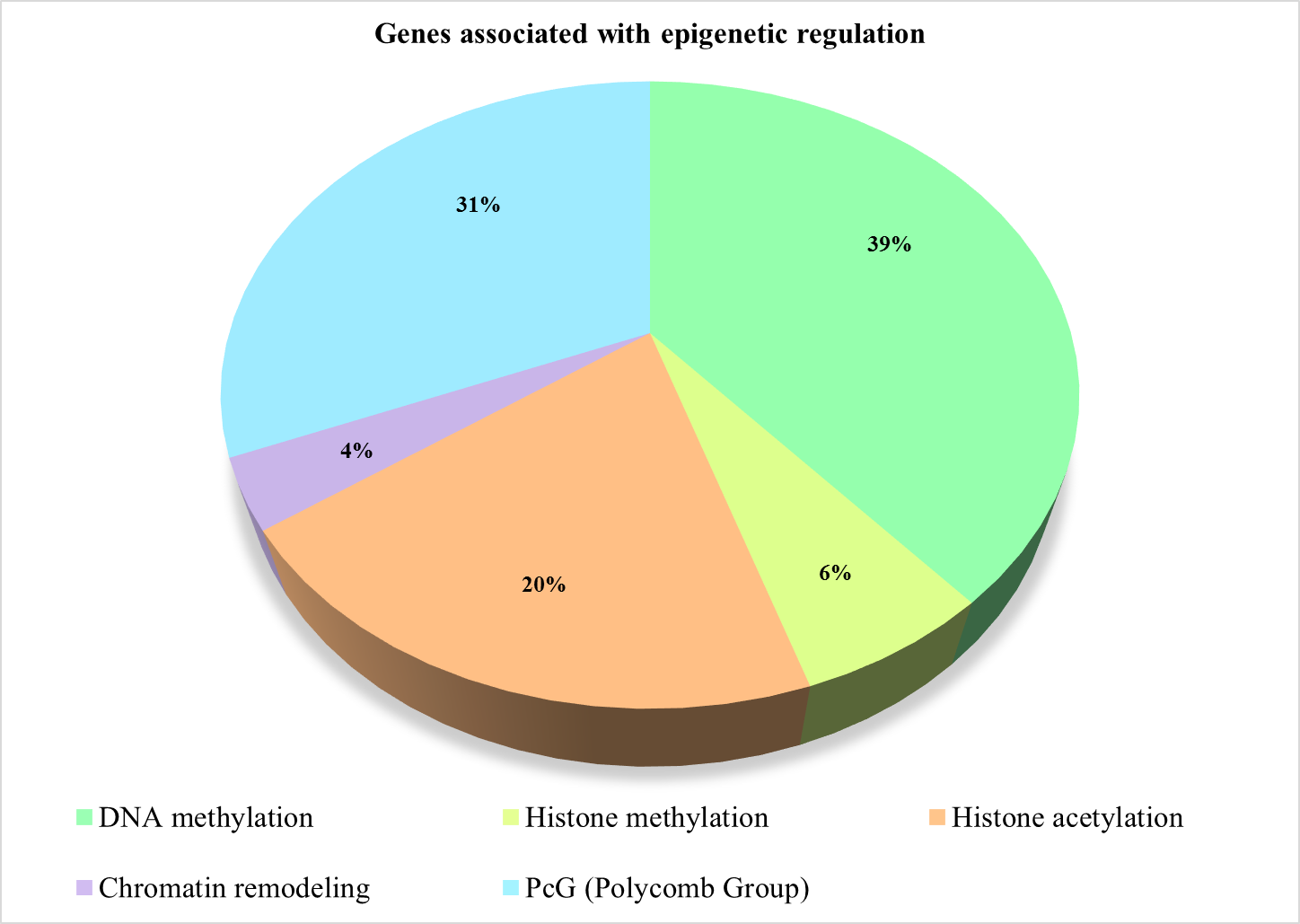

Supplement: S1 Fig — The percentage of genes associated with DNA-methylation, histone-methylation, histone-acetylation, chromatin remodeling, and polycomb group has been identified from the combined analysis of ChIP-Seq and RNA-Seq. (TIF) [file pone.0132176.s009.TIF]

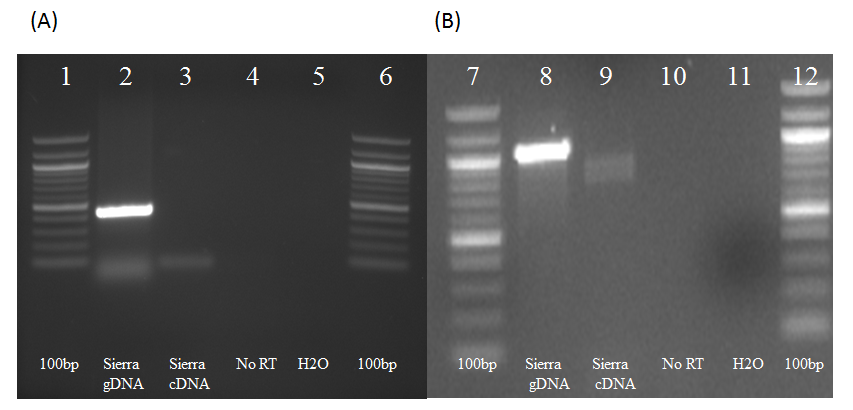

Supplement: S2 Fig — (A) Common bean marker SB, linked to the Ur-3 rust resistance locus amplify a 460 bp from genomic DNA but failed to amplify the cDNA (Lane 1: 100 bp ladder; Lane 2: SB1 gDNA; Lane 3: SB1 cDNA; Lane 4: Negative control-1 (no reverse transcriptase was added to cDNA synthesis); Lane 5: Negative control-2 (H2O only); Lane 6: 100 bp ladder). (B) Primers from NAC-transcriptional gene factor-like 9 (Phvul.010G120700) amplified intronic gDNA yielding a 963 bp amplicon from gDNA and 731 bp amplicon from cDNA. The order and contents of lanes 7 to 12 are identical to those in panel A. (TIF) [file pone.0132176.s010.TIF]

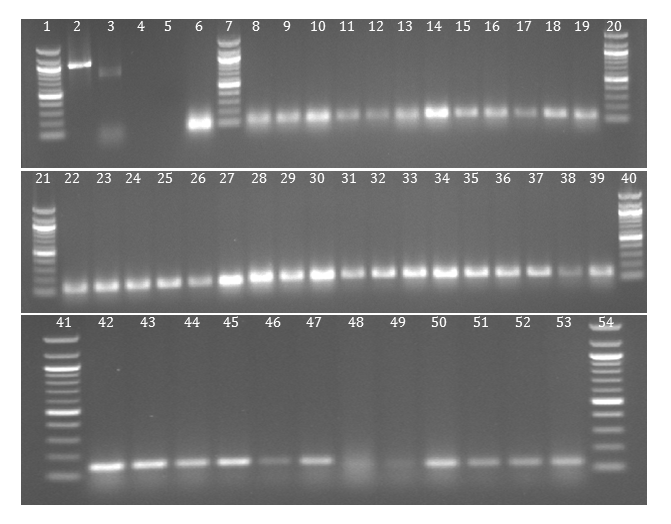

Supplement: S3 Fig — The Figure illustrates the products amplified by using RT-PCR from seven selected genes. Lane 1: 100 bp ladder; Lanes 2: gDNA of SB1; Lane 3: leaf cDNA, Lane 4: Negative control-1 (no reverse transcriptase added to SB1 cDNA), Lane 5: Negative control-2 (H2O), Lane 6: Positive control (cons7). Lanes 8–13: DREP/LRR, Lanes 14–19: Cytochrome p450, Lanes 22–27: Calmodulin, Lanes 28–33: WRKY-7 TF, Lanes 34–39: Myb like TF, Lanes 42–47: Chitinase, and Lanes 48–53: bZIP TF. Other lanes with 100 bp ladder: 7, 20, 21, 40, 41 and 54. (TIF) [file pone.0132176.s011.TIF]

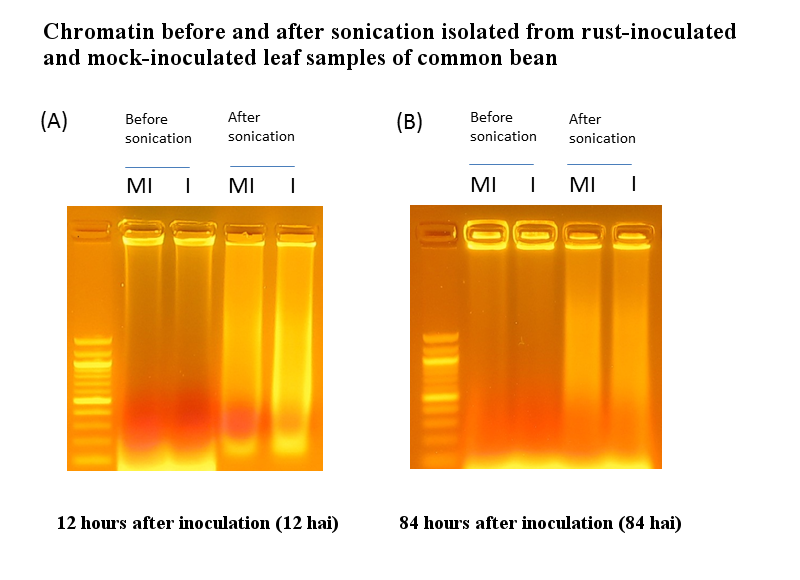

Supplement: S4 Fig — The chromatin was sonicated five times, each for a 15 s pulse on power 6 using a Soniprep, to shear DNA to approximately 100–350 bp fragments. (A) Chromatin from 12h rust-inoculated and mock-inoculated samples. Lane 1: 100 bp ladder; Lanes 2–3 mock-inoculated and inoculated samples before sonication; Lanes 4–5 mock-inoculated and inoculated samples after sonication. (B) Chromatin from 84h rust-inoculated and mock-inoculated samples. Lane 1: 100 bp ladder; Lanes 2–3 mock-inoculated and inoculated samples before sonication; Lanes 4–5 mock-inoculated and inoculated samples after sonication. (TIF) [file pone.0132176.s012.TIF]
